# Turning Disposed into Disposable—Development of Single-Use Products from Underutilized Brewery Wastes

**DOI:** 10.3390/foods15050860

**Published:** 2026-03-04

**Authors:** Aleksander Hejna, Mateusz Barczewski

**Affiliations:** Institute of Material Technology, Poznan University of Technology, Piotrowo 3, 61-138 Poznań, Poland; mateusz.barczewski@put.poznan.pl

**Keywords:** brewers’ spent grain, spent yeast, waste management, recycling, disposable plates

## Abstract

Although the market recently shifted toward low- or non-alcoholic drinks, the beer sector is an important branch of industry in Europe. It stimulates local economies and communities, thereby justifying the need for its development. Both economic and environmental benefits could be achieved through proper management of the generated by-products, enabling them to stay in a loop. Such an approach aligns with currently postulated sustainability-oriented trends. Herein, a solution for the simultaneous management of the two main by-products of beer production is described. The spent yeast (SY) was used as a potential binder for brewers’ spent grain (BSG)-based products, representing a highly innovative solution given the state of the art. Using SY without treatment or with minimal addition of common organic acids (citric, succinic, and tartaric) enabled efficient bonding of the final product. It yielded properties similar to those of commercial counterparts, with a flexural modulus exceeding 1 GPa and a flexural strength exceeding 6 MPa. Because of the nature of the applied raw materials and their inherent moisture sensitivity (water contact angle < 50°), the final product was coated with vegetable oil. The applied coating, after thermooxidation-induced crosslinking, protected against moisture and humidity (water contact angle > 80°), potentially broadening its application range. The application potential was confirmed from a technical point of view through the efficient manufacturing of disposable plates. Nevertheless, their implementation in industrial practice must be preceded by meeting proper criteria for food-contact materials related to the stability and odor of the plates and coatings and migration of their components into food products.

## 1. Introduction

Despite the substantial negative impact of the recent pandemic, the brewing industry should still be considered an essential economic sector in Europe. According to the data published by the organization, The Brewers of Europe, it generates far more than 2 million jobs in the whole value chain, “from grain to glass,” and on average, 1 job in a brewery generates a further 17 jobs in the economy [1]. Therefore, the beer sector directly affects over 1% of total European Union (EU) employment [2]. Based on the European Beer Trends—2024 Edition report [3], direct employment in the European brewing sector in 2023 was 137,712 people (data from multiple countries, like Austria, Cyprus, Czech Republic, Latvia, Lithuania, Luxembourg, Malta, Norway, Spain, Sweden, and the United Kingdom, were not included). Simultaneously, the entire beer sector is still in the recovery phase after the pandemic, which affected the entire value chain, on-trade sales, shifting consumption patterns, and caused financial losses. Despite post-pandemic inflation and rising energy prices, breweries are actively working and investing substantial amounts (in some countries, the investment level exceeds 25% of turnover [4]) to return to pre-pandemic production levels, stimulating local economies and impacting communities.

Except for the most obvious increase in demand for beer, one potential way to enhance the profitability of the brewing sector is the development of efficient methods for waste management, which could provide actual value for the generated by-products. The most substantial side streams generated during beer production are brewers’ spent grain (BSG) and spent yeast (SY), which account for ~85 and ~15 wt% of solid by-products, respectively [5]. They are currently somehow underutilized, as most breweries distribute BSG and SY among local farmers, who apply them as animal feed or soil additives. Nevertheless, such approaches are mainly aimed at the reduction of utilization costs charged by specialized waste management companies and hardly focus on using BSG and SY’s actual potential.

Numerous applications, primarily related to food or pharmaceutical sectors, involve the extraction of particular components, which is often time-consuming and generates additional side streams, e.g., due to the use of various solvents [6]. Ideally, such approaches should be implemented into more comprehensive cascade systems, which provide potential uses for all of the components of utilized wastes. However, the development of such a holistic solution requires an enormous workload and integration of multiple sectors and stakeholders, which poses a substantial limitation. On the other hand, management methods in materials science are more popular, assuming the use of wastes as a whole without generating additional by-products [7,8]. Depending on the final application, they may potentially include minor modifications related primarily to their physical state (e.g., particle size reduction or fractionation). They are typically required during the development of polymer-based composites, as smaller particles translate to a bigger interfacial area and enhanced stress transfer, directly affecting mechanical performance [9]. BSG has already been repeatedly applied as filler for polymer composites based on polypropylene [10], polyethylene [11], natural rubber [12], poly(vinyl alcohol) [13], polybutylene succinate [14,15], and poly(ε-caprolactone) [16], as well as thermoplastic starch and its blends [17,18,19]. The majority of these studies pointed to the enhancement of processing attributed to partial plasticization induced by the relatively high protein content, but also to limitations related to the interfacial hydrophilicity gap.

On the other hand, grinding of BSG can be omitted during the development of engineered wood materials, like particle board [7,20], or less demanding products, like single-use biodegradable plant pots [21] or packaging trays [22]. However, except for some examples, these materials often utilize synthetic binders, which limit their sustainability. Engineered wood materials typically contain various resins, like urea-formaldehyde or melamine derivatives, while manufacturing disposable products frequently assumes the application of, e.g., polyvinyl acetate. The application of more sustainable binders, like starch, is still somewhat limited.

Considering the other major brewery by-product, SY, its uses are mainly focused on the animal feed and biorefinery sectors, while materials science-oriented applications have been so far overlooked. The only examples have been reported in our previous studies [23,24], where SY was applied as a functional co-filler for sustainable polymer composites along with BSG and spent hops, limiting the interfacial hydrophilicity gap and facilitating melt processing due to reduced viscosity.

Nevertheless, there are obvious reasons why BSG and SY applications in materials science are limited. The literature mentions essential issues associated with their secondary uses. Due to their high moisture contents, BSG and SY utilization should be restricted locally to ensure profitability of their secondary applications, as indicated by Ben-Hamed et al. [25]. These restrictions are related to transport costs, the impact of which actually depends on the price of the final product, but also to their high moisture content and the perishability of by-products when used without drying, which is often an economic obstacle [26]. Therefore, it would be a significant advantage if developed solutions could utilize BSG and/or SY as-received without drying.

The development of an efficient solution for the utilization of beer sector by-products is in the interest not only of the brewing industry but also of the gastronomy and food service sector, which currently faces limitations in the manufacture of disposable plastic products. With the current expertise and equipment facilities of the plastics sector, disposable products could be manufactured from alternative resources, yielding environmental benefits. Herein, we proposed a novel solution for the simultaneous management of still underutilized BSG and SY. Both of these materials, which account for almost 100% of solid brewery by-products, could be used to manufacture single-use products such as dishes or packaging components. In this work, SY has been applied as-received, without an additional drying step, as a potential binder for dried BSG in the compression molding process, following our patent application [27]. Contrary to previous examples [28], SY has been applied without any modifications, which boosts the economic and ecological aspects of final products.

## 2. Materials and Methods

### 2.1. Materials

BSG and SY were obtained from two different local breweries, Lech Browary Wielkopolski (Poznań, Poland) and Browar Fortuna Sp. z o.o. (Miłosław, Poland), respectively. BSG originated from brewing light lager beer and was dried before application for 24 h at 60 °C using a Memmert ULE 500 cabinet dryer (Schwabach, Germany). Drying was conducted to avoid BSG spoilage. SY consisted of bottom-fermentation yeast and was refrigerated to inhibit its proliferation. The moisture contents of the applied raw materials, determined using a moisture analyzer AXIS BTS 100 (Gdańsk, Poland), were 10.0 and 83.5 wt%, respectively.

**Figure 1:**
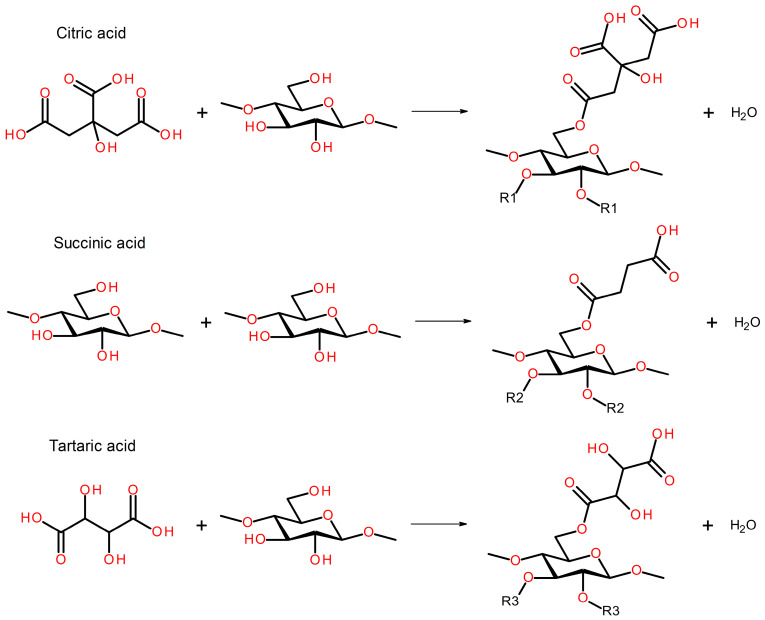
Potential chemical reactions occurring between applied brewing industry by-products and applied organic acids. R1 indicates citric acid radical, R2 indicates succinic acid radical, and R3 indicates tartaric acid radical.

**Figure 2:**
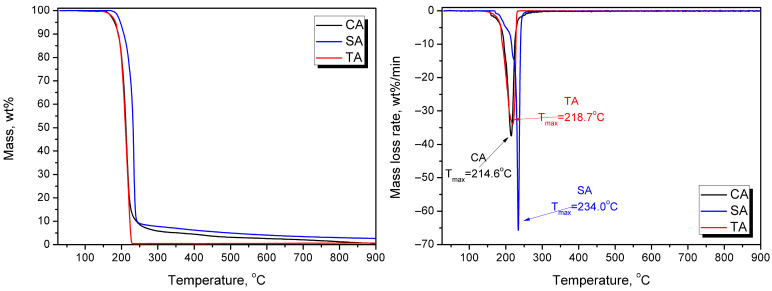
Mass loss (**left**) and derivative of thermogravimetric curves (**right**) plotted for the applied organic acids.

Citric acid (CA), succinic acid (SA), and L-(+)-tartaric acid (TA), all anhydrous analytical grade, were obtained from Chemat Adam Taszner (Gdańsk, Poland) and used as received. They were characterized with moisture contents of 0.19, 0.08, and 0.04 wt%, respectively. They were used as crosslinking agents, as reported in the literature summarized in the review by Dudeja et al. [29]. Figure 1 presents the potential chemical reactions occurring between applied brewery by-products and organic acids. Figure 2 presents the results of thermogravimetric analysis (TGA) of the applied modifiers.

Hazelnut, linseed, peanut, sesame, sunflower, and walnut oils were applied as coatings for the prepared materials. They were acquired in local grocery stores (Poznań, Poland). Table 1 presents basic information and literature-based composition data. Dynamic viscosity was assessed using an Anton Paar MCR 301 cone-plate rheometer (Graz, Austria) with a 25 mm cone at 30 °C. The results presented in the table are the average values of 600 s of measurement performed at a constant shear rate of 100 s^−1^.

### 2.2. Manufacturing of BSG/SY Materials

Initially, both BSG and SY, with the optional addition of a particular organic acid, were homogenized using a GL 4219 planetary mixer from Gerlach (Drzewica, Poland). Mixing was conducted at room temperature for 3 min. Then, a proper amount of material (calculated based on the desired density of the final product) was introduced into the metal mold and compressed using a Fontijne LabManual 300 (Rotterdam, The Netherlands) laboratory hydraulic press. Various processing times, temperatures, and pressures were investigated. Table 2 presents the compositions and processing parameters of the developed materials. For more detailed information, Table S1 provides their formulations on a dry basis.

### 2.3. Thermooxidation of Vegetable Oils

To assess the potential of the selected vegetable oils as coatings for the developed materials, their thermooxidation and related crosslinking were investigated. For the preliminary analysis, approximately 10 g of the chosen oil was placed on a glass Petri dish in a laboratory oven preheated to 120 °C. Samples were removed at 3, 7, and 14 days to assess thermooxidation-induced changes. After a preliminary evaluation, selected oils were subjected to similar treatments at 80 °C and 100 °C.

### 2.4. Coating of Developed Materials with Vegetable Oils

To analyze the protective impact of the thermooxidized vegetable oils, they were applied as coatings to the developed materials. Samples selected based on the structural and mechanical property analysis were coated with the chosen oils using a hand lay-up procedure. Sample surfaces were uniformly coated with a similar, pre-weighed amount of oil using a paintbrush. Then, samples were placed in a laboratory oven preheated to 100 or 120 °C and held for 24 h.

### 2.5. Evaluation of Industrial Potential

To assess the industrial potential of the developed materials, selected formulations were used to manufacture disposable plates. The manufacturing process was conducted similarly to that during the laboratory tests. Initially, both BSG and SY were homogenized using a GL 4219 planetary mixer from Gerlach (Drzewica, Poland). Mixing was conducted at room temperature for 3 min. Then, a proper amount of material (calculated based on the desired density of the final product) was introduced into the metal mold and compressed using a PH-90 laboratory hydraulic press from P.U.H. Nysa (Nysa, Poland). Table 3 presents the compositions and processing parameters of the developed materials, which were selected after preliminary trials. Variations compared to the parameters presented in Table 2 were related to the more complex shape of the final product and different characteristics of the applied mold, which required significantly higher pressure for proper filling. For more detailed information, Table S2 provides the formulations on a dry basis.

### 2.6. Characterization

The appearance of the developed materials and oils applied as modifiers was captured using a SONY (Tokyo, Japan) RX100 VI digital camera. Each sample was placed in a light tent to enhance image quality.

The apparent density of samples was calculated in accordance with ISO 845:2006 [49], as a ratio of the sample weight to the sample volume (g/cm^3^). The rectangular samples were measured using a slide caliper (accuracy 0.1 mm) and weighed using an electronic analytical balance (accuracy 0.0001 g).

The developed materials were analyzed using an Anton Paar Ultrapyc 5000 Foam gas pycnometer (Graz, Austria). The following measurement settings were applied: gas—nitrogen; target pressure—19.0 psi; temperature control—on; target temperature—20.0 °C; flow mode—coarse powder; cell size—10 cm^3^; preparation mode—pulse, 4 times. Based on the pycnometric analysis, the porosity of materials was calculated according to Equation (1):

Porosity = (V_geo_ − V_pyc_)/V_geo_ × 100% (1)

where: V_geo_—geometric volume of the sample measured with a slide caliper, cm^3^; V_pyc_—volume of the sample determined with a gas pycnometer, cm^3^.

The flexural strength of the composites was measured according to ASTM D790-17 [50]. The beam-shaped samples, with dimensions of 4 × 10 × 100 mm^3^, were measured using a slide caliper with an accuracy of 0.1 mm. The bending test was performed using a Zwick/Roell Z020 model 5101 universal testing machine (Ulm, Germany) at 25 °C, 30% relative humidity, and a constant crosshead speed of 10 mm/min.

Shore hardness type D was measured using a Zwick 3131 durometer from Zwick Roell (Ulm, Germany) according to ISO 868:2003 [51]. Each evaluation was prepared for five test specimens.

Surface wettability was determined through static water contact angle (WCA) measurements using an Ossila L2004 contact angle goniometer (Sheffield, UK) equipped with a camera and Ossila Contact Angle v 3.0.9.1 software. Ten contact angle measurements were taken at random positions by placing drops of ~1 µL of distilled water on the sample surface using a dedicated syringe. The average values were calculated and reported.

Color coordinates in the CIELab space were determined for the samples using an NR145 colorimeter from Envi Sense (Poland) operating in a 45°/0° geometry. The total color change ΔE, was calculated according to Equation (2):

ΔE = ((ΔL)^2^ + (Δa)^2^ + (Δb)^2^)^0.5^ (2)

where: ΔL, Δa, and Δb indicate differences in the L*, a*, and b* parameters determined for as-received and aged samples, respectively.

Fourier transform infrared spectroscopy (FTIR) was performed using a Jasco (Tokyo, Japan) FT/IR-4600 apparatus in attenuated total reflectance (ATR) mode. FTIR analyses were performed with 128 scans at a resolution of 4 cm^−1^ over the wavenumber range of 4000–400 cm^−1^.

The thermal properties of the samples were measured through differential scanning calorimetry (DSC) using a DSC 204 F1 Phoenix^®^ apparatus from Netzsch (Selb, Germany). Measurements were performed on 8 ± 0.5 mg samples placed in aluminum crucibles with pierced lids in the temperature range of −60 to 100 °C, under a nitrogen atmosphere, at a heating/cooling rate of 10 °C/min.

## 3. Results and Discussion

### 3.1. Manufacturing and Appearance of BSG/SY Composite Materials

As shown in Table 2, the initial composition of the input material for compression molding was 3:1 BSG:SY, yielding a cumulative moisture content of 28.4 wt%. Such a composition does not reflect the actual ratio of BSG to SY generation in breweries, which, according to multiple reports, is around 5.7:1, but it was selected as a starting point to ensure sufficient moisture content to bind the material during compression processing. Based on the final material’s targeted density, the input mass was determined. The volume of the mold applied during our laboratory work was 48.4 cm^3^, and the input mass was 30 g, yielding a targeted density of 620 kg/m^3^.

**Figure 3:**
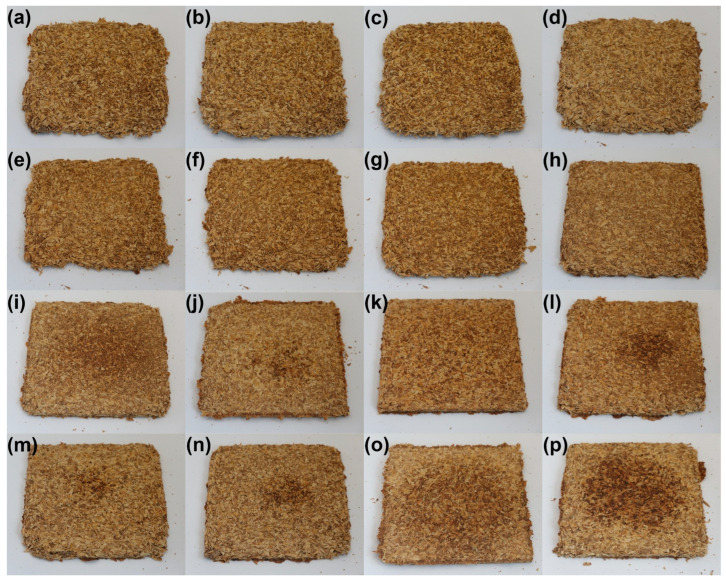

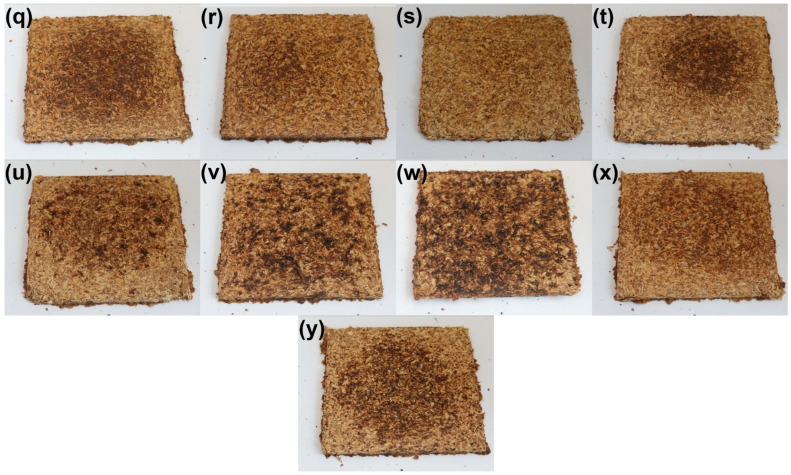
The appearance of developed BSG/SY samples according to the formulations provided in Table 2 (all formulations presented according to the template BSG/SY ratio, organic acid type and content—not mentioned for samples without addition, targeted density in kg/m^3^, molding temperature in °C, molding pressure in bar, and molding time in s): (**a**)—75/25, 620, 200, 20, 10; (**b**)—75/25, 620, 200, 20, 20; (**c**)—75/25, 620, 200, 20, 30; (**d**)—75/25, 620, 200, 20, 60; (**e**)—75/25, 620, 200, 20, 120; (**f**)—75/25, 620, 200, 20, 180; (**g**)—75/25, 723, 200, 20, 120; (**h**)—75/25, 826, 200, 20, 120; (**i**)—75/25, 930, 200, 20, 120; (**j**)—85/15, 930, 180, 20, 30; (**k**)—85/15, 930, 200, 20, 30; (**l**)—85/15, 930, 220, 20, 30; (**m**)—85/15, 930, 200, 25, 30; (**n**)—85/15, 930, 200, 30, 30; (**o**)—85/15, 1240, 180, 20, 30; (**p**)—85/15, 1240, 200, 20, 30; (**q**)—85/15, 1240, 220, 20, 30; (**r**)—92.5/7.5, 1240, 200, 20, 30; (**s**)—92.5/7.5, 1240, 200, 25, 30; (**t**)—92.5/7.5, 1240, 200, 30, 30; (**u**)—82.87/14.63, CA 2.5, 1240, 180, 20, 30; (**v**)—80.75/14.25, CA 5.0, 1240, 180, 20, 30; (**w**)—76.50/13.50, CA 10.0, 1240, 180, 20, 30; (**x**)—80.75/14.25, SA 5.0, 1240, 180, 20, 30; (**y**)—80.75/14.25, TA 5.0, 1240, 180, 20, 30.

Figure 3 presents the appearance of the developed samples. For the lowest values of targeted density (Figure 3a–f—620 kg/m^3^, Figure 3g—723 kg/m^3^, and Figure 3h—826 kg/m^3^), evident shortages in sample corners can be observed, which point to insufficient mass input relative to the mold size. It indicated that, due to a lack of material flow during molding, the mold was not efficiently filled, and the product was not manufactured with the desired density, irrespective of the analyzed compression time. Elongating the compression time improved surface quality and reduced roughness. Moreover, surface darkening was noted, which was attributed to the prolonged impact of heat on the processed material, which might cause partial degradation of the material and induce non-enzymatic browning reactions (NEBRs) [52].

For a processing time of 120 s, the impact of input mass (and hence targeted density) was investigated. Figure 3e,g–i present the appearance of samples with densities of 620, 723, 826, and 930 kg/m^3^, respectively. It can be seen that mold filling precision was significantly improved, which also affected surface quality and reduced roughness. Therefore, further work was conducted to achieve a targeted density of at least 930 kg/m^3^.

At the same time, the good coherence of the material with the highest mass input enabled the share of SY applied as a potential binder in the analyzed composition to be reduced to 15 wt%. Such a mass ratio of BSG and SY (85/15) aligns with multiple literature reports related to the generation of these by-products on an industrial scale [53,54,55], thereby enhancing the application potential of the developed materials. Moreover, reducing the share of SY yielded a lower cumulative moisture content of the analyzed material, 21.0 wt% compared to 28.4 wt%, which enabled the processing time to be shortened to 30 s.

Comparison of the samples presented in Figure 3i,k indicated that increasing the BSG share enabled more precise filling of the applied mold, which was associated with a higher portion of fibrous BSG particles. Shortening the compression time resulted in a more homogeneous surface appearance, which should beneficially impact the overall quality of the final products [56].

For a targeted density of 930 kg/m^3^ and a compression time of 30 s, the impacts of compression temperature (Figure 3j–l) and pressure (Figure 3k,m,n) were analyzed. Considering the temperature, it can be seen that the appearance of the final products differed, which was attributed to NEBRs resulting in melanoidins production, which have been repeatedly reported as a natural coloring agent [57]. Considering the compression pressure, its impact on product appearance was negligible. However, regardless of the selected parameters, the filling degree of the applied mold remained insufficient; therefore, further tests were performed at a targeted density of 1240 kg/m^3^. Comparison of Figure 3j–l with Figure 3o–q shows the beneficial impact on mold filling, yielding higher dimensional precision, enhancing the appearance of the final product. Considering the surface appearance, the browning resulting from NEBRs was significantly more pronounced at the higher targeted density, which can be regarded as beneficial, as melanoidins, aside from their color features, contribute to reduced hydrophilicity—a vital aspect of multiple disposable products [58].

Based on these processing parameters, two further modifications were applied. The first one was related to an additional increase in the BSG share to 92.5 wt% and a reduction in the SY share to 7.5 wt% (Figure 3r–t). It can be seen that the effect was similar to that observed in previous composition adjustments (Figure 3i,k), with slightly better mold filling and more pronounced browning.

The other modification involved applying various organic acids as potential crosslinking agents. Such an approach has been repeatedly reported for cellulose-based materials [59,60]. In the present study, citric, succinic, and tartaric acids were applied in proportions based on previous results [61]. Based on the aforementioned analysis, the following processing parameters were selected for organic acid modification: targeted density of 1240 kg/m^3^, processing time of 30 s, pressure of 20 bar, and temperature of 180 °C. The reduction in molding temperature was associated with the thermal stability of applied modifiers (see Figure 2) and ensured their efficiency. Such an effect should be considered highly auspicious for the sustainability of the developed materials, given the lower energy consumption of the manufacturing process. Based on the literature [28,60], three CA contents were analyzed: 2.5, 5, and 10 wt%. Increasing the CA share led to significant surface discoloration, likely due to the potential decomposition of the modifier (primarily dehydration starting at 175 °C) and the generation of aconitic acid [62]. Moreover, increasing its content led to surface stickiness, which is considered significantly undesirable. A similar effect, albeit less intense, was observed for TA, which also releases water [63]. It was considerably less pronounced for SA, which was attributed to the lack of hydroxyl groups and higher thermal stability [64].

### 3.2. Physico-Mechanical Structure of BSG/SY Composite Materials

**Figure 4:**
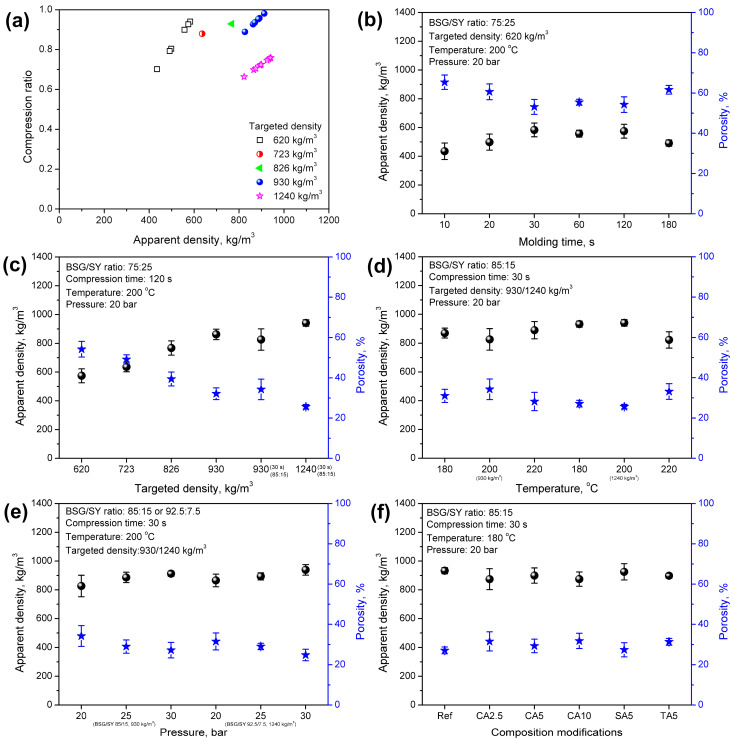
Plots of (**a**) dependence of compression ratio on targeted density; (**b**) impact of molding time on apparent density and porosity of prepared materials; (**c**) impact of targeted density on apparent density and porosity of prepared materials; (**d**) impact of molding temperature on apparent density and porosity of prepared materials; (**e**) impact of molding pressure on apparent density and porosity of prepared materials; and (**f**) impact of composition modifications on apparent density and porosity of prepared materials. In (**b**–**f**), black balls indicate apparent density, while blue stars indicate porosity.

**Figure 5:**
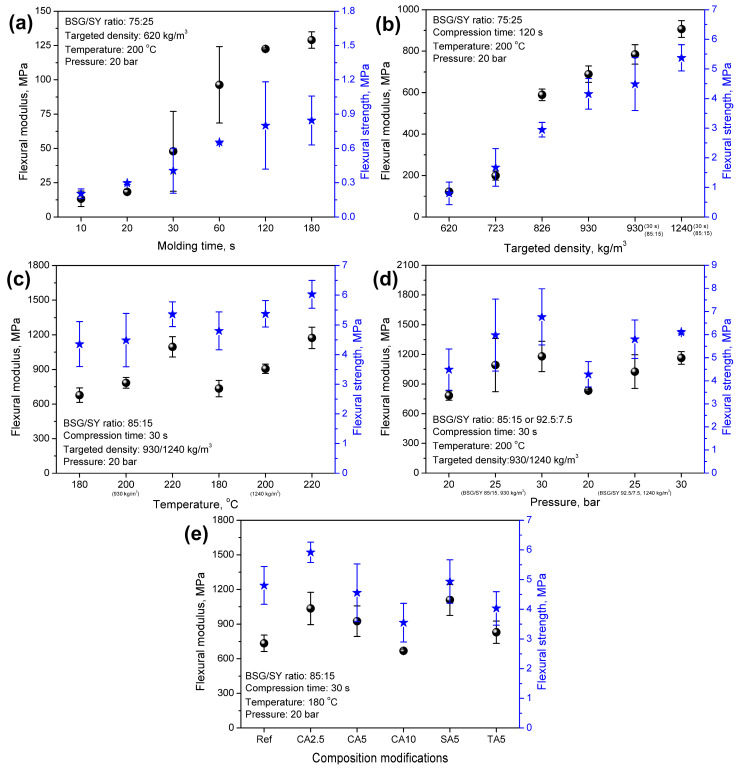
The impact of (**a**) molding time, (**b**) targeted density, (**c**) molding temperature, (**d**) molding pressure, and (**e**) composition modifications on the flexural modulus and strength of developed materials. Black balls indicate flexural modulus, while blue stars indicate flexural strength.

**Figure 6:**
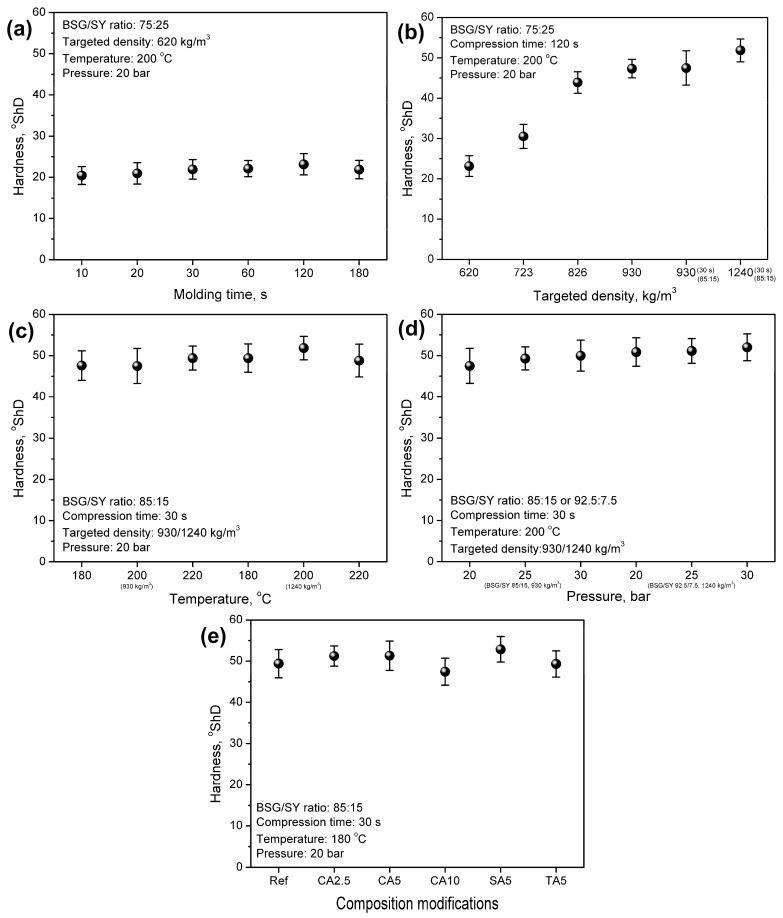
The impacts of (**a**) molding time, (**b**) targeted density, (**c**) molding temperature, (**d**) molding pressure, and (**e**) composition modifications on the hardness of developed materials.

Figure 4 presents the impacts of the applied processing parameters and composition modifications on the apparent density and porosity of the developed materials. Figure 5 and Figure 6 show their implications on mechanical performance, which is vital for potential applications. The results of the 3-point bending test and hardness analysis mirrored the observations made during visual inspection of the samples and aligned with the apparent density variations.

It can be seen that all of the developed materials showed an apparent density lower than the targeted density, which is quite typical for engineered wood materials consisting of wood or wood-like particles and a synthetic binder [65,66]. In the presented case, the compression ratio was clearly driven by the applied processing parameters. For the lowest targeted density of 620 kg/m^3^, the apparent density ranged from 435 to 583 kg/m^3^, yielding a compression ratio of 0.70–0.94. An increase in density enhanced the flexural modulus and strength, as well as the hardness of the developed materials. Such an effect was associated with an elongation of molding time, which enabled more efficient particle compaction, enhanced moisture evaporation from SY, and a greater extent of NEBRs, whose products could act as potential binders for the developed materials [67]. On the other hand, excessive elongation to 180 s reduced the density and compression ratio, thereby enhancing porosity, which could be related to the decomposition of brewery by-products. Considering mechanical performance, no significant changes were noted for molding times exceeding 120 s. Despite maintaining its flexural properties, the material’s hardness was reduced, which could be attributed to enhanced porosity [68].

The gradual strengthening of materials was induced by increasing the mass input during compression molding, thereby increasing the targeted density. Figure 4b shows that a 50% increase in density from 620 to 930 kg/m^3^ resulted in a more than 5.5-fold increase in the flexural modulus and a 5.2-fold increase in flexural strength. Moreover, the hardness was doubled, increasing from 23.16 to 47.32 ShD. Such an effect was clearly induced by the improved packing of the material within the mold and its lower porosity (see Figure 4).

Moreover, following the appearance changes, shifting the BSG/SY ratio from 75:25 to 85:15 for a similar targeted density (930 kg/m^3^) enabled maintaining the density and porosity, while simultaneously strengthening the developed materials due to the higher share of fibrous BSG, which is more effective in stress transfer. Such effects were noted despite the significant reduction in molding time from 120 to 30 s. A further increase in the targeted density to 1240 kg/m^3^ followed the aforementioned trend, associated with higher mass input, increased apparent density, reduced porosity, and enhanced mechanical performance of the materials. Notably, the enhancement of flexural properties was observed, despite a less pronounced increase in apparent density and a reduction in porosity, as indicated by the lower compression ratio for the targeted density of 1240 kg/m^3^ compared to 930 kg/m^3^ (Figure 4). This effect was confirmed by the thicknesses of the prepared samples. For a targeted density of up to 930 kg/m^3^, the thickness ranged within 3.872–4.102 mm (only 1 material, sample 10 deviated with a thickness of 4.432 mm), while for 1240 kg/m^3^, the thickness ranged within 4.080–4.648 mm, which noticeably exceeded the mold thickness (4 mm). This highlighted the need to apply significantly higher pressure during compression to fit the input material into the mold.

The analysis of compression temperature revealed a beneficial impact on mechanical performance, despite only minor changes in apparent density and porosity. Such an effect could be attributed to the more efficient moisture evaporation and higher extent of NEBRs, whose products might act as binders, enhancing the material’s cohesion [67]. Such an effect aligned with the results presented later in the surface wettability analysis.

A beneficial impact on mechanical performance was also noted for the applied pressure. Given the lack of material flow in the mold (in contrast to, e.g., polymer-based materials processed in the molten state), pressure played a crucial role in enhancing the compression ratio of the developed materials. For the targeted density of 930 kg/m^3^, an increase in pressure from 20 to 30 bar increased the compression ratio from 0.89 to 0.98, while for the targeted density of 1240 kg/m^3^, a rise from 0.70 to 0.76 was observed. As presented in Figure 5 and Figure 6, the flexural properties and hardness were beneficially affected by these structural changes. Enhanced packing of the material within the mold significantly improved stress-transfer efficiency and increased the material’s stiffness and strength.

The introduction of organic acids into material formulations also showed a beneficial impact on flexural performance. Compared to the reference sample, prepared using a compression temperature of 180 °C, stiffening of materials, expressed by a rise in the flexural modulus, was noted, which pointed to efficient bonding of the material, as these improvements occurred despite the porosity increase related to the higher amount of evaporated water generated in the reactions between organic acids and the applied brewery by-products (Figure 1). However, increasing CA loading reduced the mechanical performance. Such an effect could be attributed to the plasticizing effect induced by residual CA and has already been reported in cellulose-based materials crosslinked with CA [69,70,71]. On the other hand, the application of 5 wt% of SA and TA showed superior effects to CA due to limited plasticization. This was associated with a lower number of carboxyl groups, which could effectively react with the hydroxyl and amine groups present in the structures of BSG and SY. Comparison of the bicarboxylic acids revealed the superior performance of SA, which was related to the longer hydrocarbon chain and less steric hindrance caused by the hydroxyls present in the structure of TA.

### 3.3. Surface Wettability of BSG/SY Composite Materials

**Figure 7:**
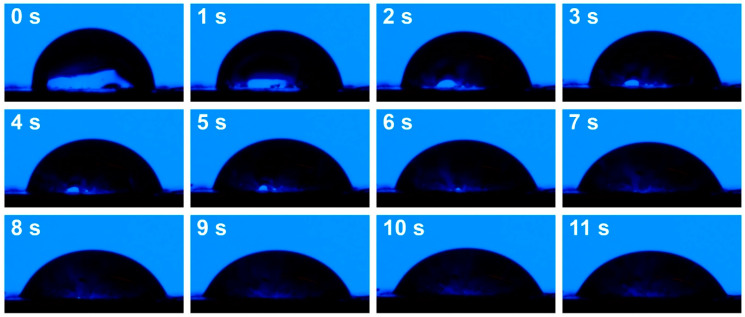
Exemplary images of sample 11 (930 kg/m^3^, 200 °C), illustrating the absorption of the water drop by the material’s surface.

**Figure 8:**
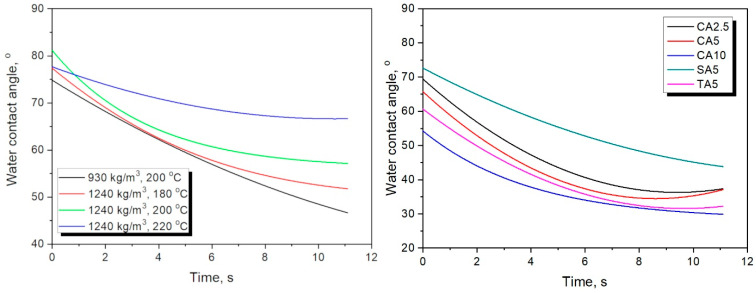
The changes in water contact angle for the analyzed samples over time plotted for (**left**) various targeted densities and molding temperatures, and (**right**) various composition modifications.

Based on the above results, selected samples were investigated for their water resistance. Such an analysis was conducted using a goniometer, and resistance was assessed by changes in WCA over time. The initial study was conducted for 10–12 s, which, based on preliminary results, was long enough to assess WCA changes. Figure 7 presents exemplary images of sample 11 (930 kg/m^3^, 200 °C), illustrating the absorption of the water drop by the material’s surface, while Figure 8 presents the changes in WCA for the analyzed samples.

Figure 8 presents the impact of the material’s targeted density and compression temperature. Increasing the mass input rate limited the drop penetration rate, which aligned with the changes in apparent density and porosity. Higher porosity in the sample, characterized by a targeted density of 930 kg/m^3^, enhanced capillary water penetration, similar to various porous materials, such as particleboard [72]. Considering the impact of the molding temperature, it did not affect density and porosity in a straightforward manner (Figure 4). Nevertheless, it clearly reduced the surface hydrophilicity of the developed materials, which was attributed to NEBRs and their products, repeatedly reported as moderately hydrophobic due to the glycation of amino-bearing compounds with reducing sugars [73].

Furthermore, the impact of introducing organic acids was assessed. It can be clearly observed that, despite the beneficial impact of strengthening adhesion between particles within the developed materials, surface hydrophilicity was significantly enhanced. Such an effect could be attributed to the presence of functional groups strengthening the affinity toward water and the slightly increased porosity of materials, facilitating water penetration. Considering potential applications of the developed materials in the packaging sector or as disposable tableware, such an effect should be significantly diminished. Therefore, in the following sections, various types of vegetable oils were applied as modifiers to shift the surface character toward hydrophobicity.

### 3.4. Analysis of Oil Crosslinking

For preliminary screening, all analyzed oils were subjected to thermal treatment at 120 °C. Table 4 presents the visual changes during treatment, which were quantitatively assessed by colorimetric analysis. The results are summarized in Table 5.

**Figure 9:**
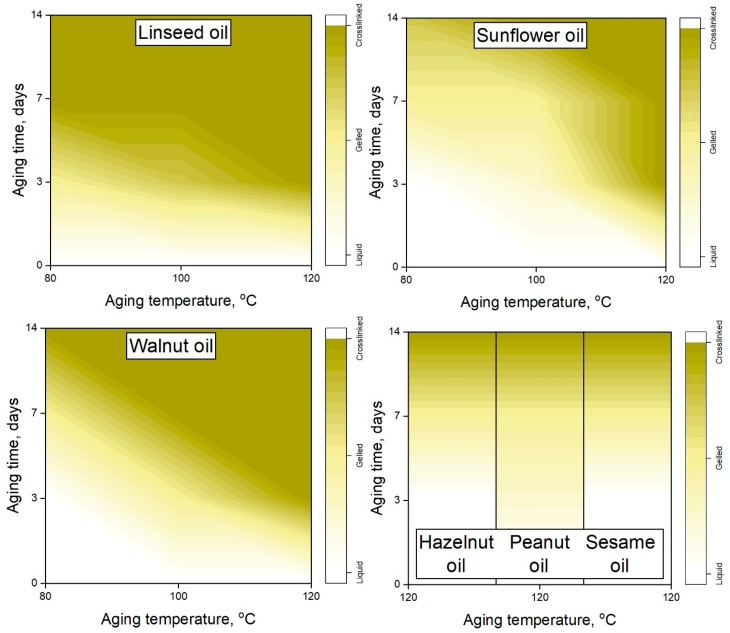
Changes in the physical state of the oils after treatment. For linseed, sunflower and walnut oils, the impacts of time and treatment were investigated.

The presented images indicate significant oxidation-induced changes in the applied oils, as previously reported in the literature [74,75] and further confirmed by FTIR and DSC analyses. Changes in the physical state of the oils after treatment were also visually assessed and are summarized in Figure 9. Based on the presented results, linseed, sunflower, and walnut oils were selected as suitable for treatment at lower temperatures (80 and 100 °C). Peanut, sesame, and hazelnut oils crosslinked only after 14 days, whereas shorter times (3 or 7 days) resulted only in partial gelling. Considering the economic aspects of potential production, the long time required for efficient modification excluded the application of hazelnut, peanut, and sesame oils. Therefore, Table 6 only presents the appearance of linseed, sunflower, and walnut oils after treatment at 80 and 100 °C.

**Figure 10:**
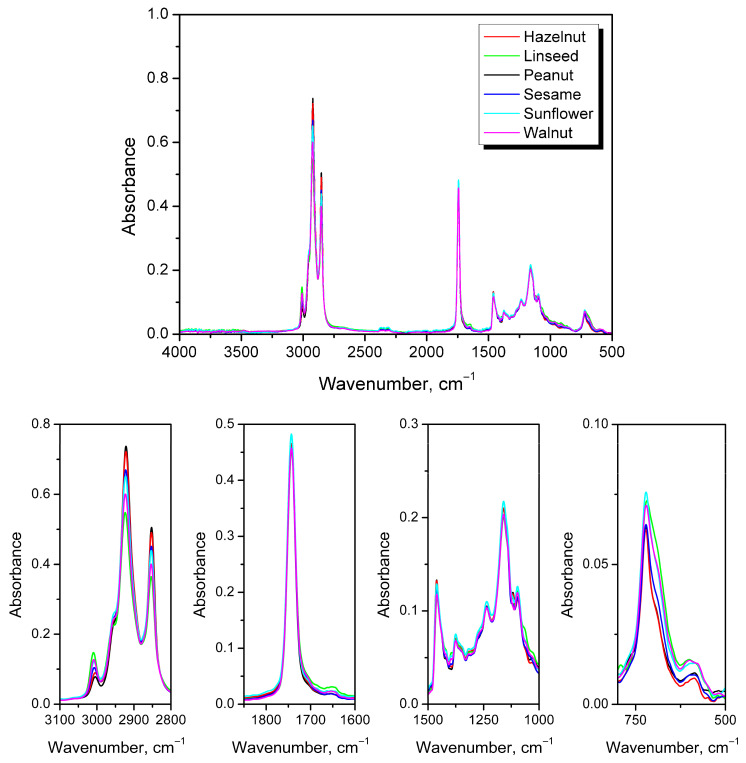
FTIR spectra of as-received oils with magnified areas pointing to the most significant structural differences.

To track changes in oil structure, FTIR spectroscopy was performed. Figure 10 presents a comparison of the FTIR spectra for all applied oils. Qualitatively, all applied oils exhibited a similar structure, consistent with the literature [76]. However, deeper investigation revealed variations in the magnitudes of specific peaks related to chemical composition, particularly the fatty acid content. Contrary to the aged oils’ spectra presented later, as-received samples did not show peaks associated with the stretching of hydroxyl groups around 3470 cm^−1^, indicating a lack of, or very low levels of, oxidation [77]. Further, a significant variation in the 2800–3100 cm^−1^ range was observed, attributed to differences in fatty acid composition [78]. The magnitudes of the peaks at 2852 and 2921 cm^−1^, resulting from the symmetric and asymmetric stretching vibrations of aliphatic CH_2_ groups, were inversely proportional to the magnitudes of the C=C bond-related peak at 3008 cm^−1^ and the related peak at 722 cm^−1^, which is typically noted for vegetable oils [79]. The latter peaks were characteristic of the stretching vibrations of *cis* double C=C bonds and were directly related to the degree of saturation [80]. Their intensities were noticeably higher for linseed, sunflower, and walnut oils, which aligned with the polyunsaturated fatty acid content presented in Table 1. The distinct signal at ~1740 cm^−1^, attributed to C=O stretching vibrations, showed similar intensity across all analyzed oils. Similar observations were made in the fingerprint region (1000–1500 cm^−1^), including peaks associated with C-H and C-O vibrations.

**Figure 11:**
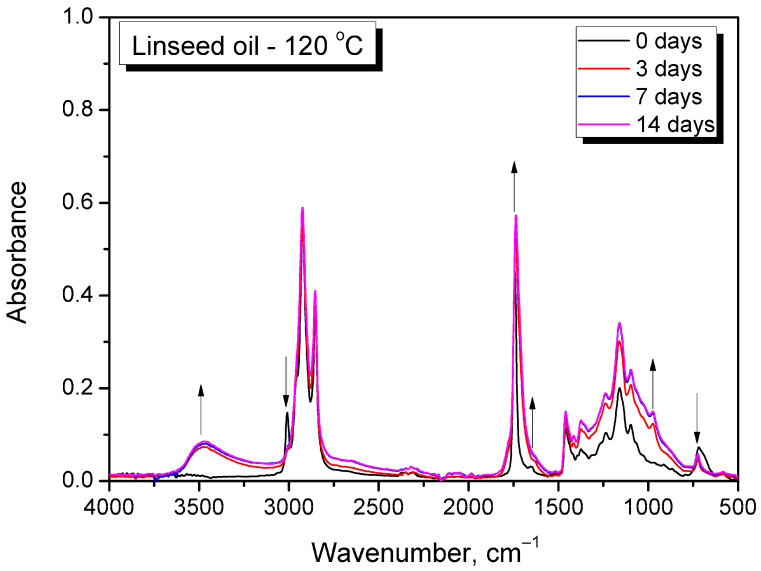
FTIR spectra of linseed oil subjected to thermooxidation at 120 °C. Arrows indicate the trend in the signal intensity changes with thermooxidation time.

Further, all oils were subjected to thermal treatment, and changes in chemical structure were investigated using FTIR spectroscopy. Figure 11 presents exemplary spectra of linseed oil treated at 120 °C, while Figures S1–S11 present the spectra of all oils treated at 120 °C and selected oils subjected to lower temperatures of 80 and 100 °C. In all cases, similar spectral changes were observed, confirming efficient oxidation. The most significant change was the appearance of a broad peak in the 3200–3600 cm^−1^ region, associated with the formation of hydroperoxides during oxidation by hydrogen-atom abstraction from unsaturated fatty acids [81]. The most significant increase in the hydroperoxide band was observed for linseed and sunflower oils, while a less pronounced increase was especially evident for peanut and hazelnut oils, which aligned with the unsaturation level indicated by the literature data (Table 1) and intensity of the 3008 cm^−1^ signal. Further, hydroperoxides decompose into secondary oxidation products, e.g., aldehydes and ketones, contributing to the significant increase in the magnitude of the distinct signal at ~1740 cm^−1^, as well as its broadening, related to the introduction of overlapping bands at 1705 and 1725 cm^−1^ and the increase in the intensity of the 1650 cm^−1^ signal [82]. Such an effect was observed for all analyzed oils. Except for the formation of hydroperoxides, the cis-to-trans rearrangement of C=C bonds is typical of oil oxidation. Such a change was also clearly indicated in the presented FTIR spectra and was expressed by the decrease in the magnitudes of the 3008 and 722 cm^−1^ peaks relative to that of the 967 cm^−1^ signal [83]. The observed variations in particular spectra and their rates aligned with the visual observations and changes in the physical state of oils subjected to thermal aging.

**Figure 12:**
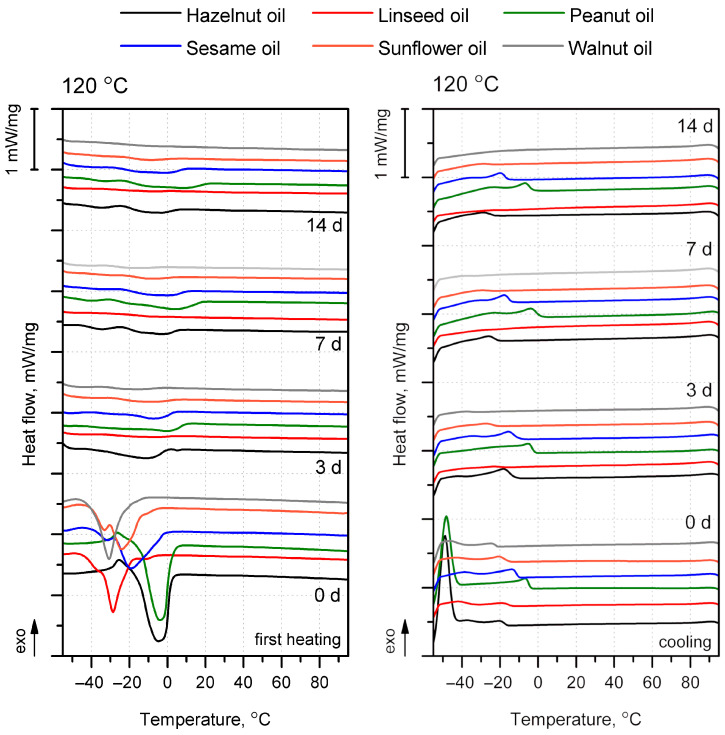
First heating (**left**) and cooling (**right**) curves of oils subjected to exposure at 120 °C for 0, 3, 7, and 14 days.

DSC has been widely used to study edible oils and has become a useful tool for assessing degradation phenomena caused by oxidation [84]. Thanks to the analysis of heat flow curves during heating and cooling, it is possible to infer indirect changes in the chemical structures of individual oil components, such as fatty acids and triglycerides [85]. Figure 12 shows the DSC curves obtained during the first heating and cooling of oils exposed to 120 °C in an oxidizing atmosphere for 0, 3, 7, and 14 days. DSC curves for all materials, including tests on sunflower, linseed, and walnut oils at various temperatures, are summarized, together with a table of detailed calorimetric data, which are presented separately in the Supplementary Materials. At the same time, the complexity of the phenomena and the multitude of overlapping thermal events limit DSC’s use to a complementary qualitative tool for assessing oil oxidation. In general, the obtained calorimetric data were consistent with the literature results for raw and oxidized oils of the same origin [86,87,88,89,90].

In the qualitative analysis of changes in the structure of edible oils caused by oxidation, differences are observed during both heating and cooling. The DSC heating curves for the oils used, not exposed to elevated temperatures or oxidative conditions, showed varying behaviors related to the presence of unsaturated triacylglycerol (TAG) [89], manifesting as single or double endothermic events. For some oils (hazelnut, peanut, and sesame), a noticeable exothermic effect preceded the endothermic effect. Chiavaro et al. [89] attributed this phenomenon to the crystallization of the TAG fraction, which did not undergo solidification during the preliminary cooling, or to the reorganization of polymorphic crystals into more stable forms, which subsequently melted. In the DSC analysis, only hazelnut and peanut oils showed distinct exothermic peaks below −40 °C, which could be attributed to the crystallization of highly unsaturated TAG. However, after just 3 days of heating, they disappeared. In all curves, additional low-intensity exothermic peaks were observed, which may have reflected the crystallization of more saturated TAG. The influence of temperature exposure shifted the peaks toward lower temperatures, with only a minimal effect on their intensity. The phenomenon of thermally induced oxidation in this case could be explained by the increase in the number of polar molecules resulting from the oxidative breakdown of lipids, which significantly limit TAG crystallization [85,91]. Depending on the oil type, in addition to those mentioned for hazelnut and peanut oils, exothermic peaks of lower intensity were observed, defined as high- and medium-temperature, the presence of which corresponded to disaturated–monounsaturated and monosaturated–diunsaturated TAGs [92]. With extension of the thermooxidation process at 120 °C, a gradual shift toward lower high-temperature peak values (below −30 °C) and the disappearance of the medium-temperature phenomenon were observed (Figures S12–S23). Prolonged treatment of oils caused the thermal effects to disappear and their intensity to decrease, both during heating and cooling. The decrease in crystallization temperature with increasing thermooxidation time and temperature (Table S3) was attributed to partial cleavage of TAG and the formation of polar compounds absorbed into the forming TAG crystal lattice, or to the formation of irregular crystal structures [85,93].

For the material series for which a significant change in the gel-like physical structure was observed (Table 6), additional tests were conducted at lower temperatures of 80 and 100 °C, the results of which are presented in the Supplementary Materials. However, no significant relationship was observed between the series aged at different temperatures regarding changes in temperature within the analyzed range, indicating that exposure time was the dominant factor influencing oil oxidation. Oils for which a significant change in physical structure was observed were characterized by a reduced intensity and temperature of the endothermic phenomenon noted during the first heating. Therefore, the potential for crosslinking in the oil, the deliberate change in its physical stability at ambient temperature after aging at elevated temperatures, and the possibility of its use as a coating for biomass-based products required a reduced proportion of low-unsaturated TAG. Using the disappearance of thermal phenomena and the flattening of the curves with increasing exposure time to an oxidative atmosphere at elevated temperatures as criteria for assessing oxidation degree, the highest oxidation degree was observed in linseed, walnut, and sunflower oils, respectively. Therefore, these oils were the most promising as coating materials and were used in the next stage of the study.

### 3.5. Surface Modification by Oils

**Figure 13:**
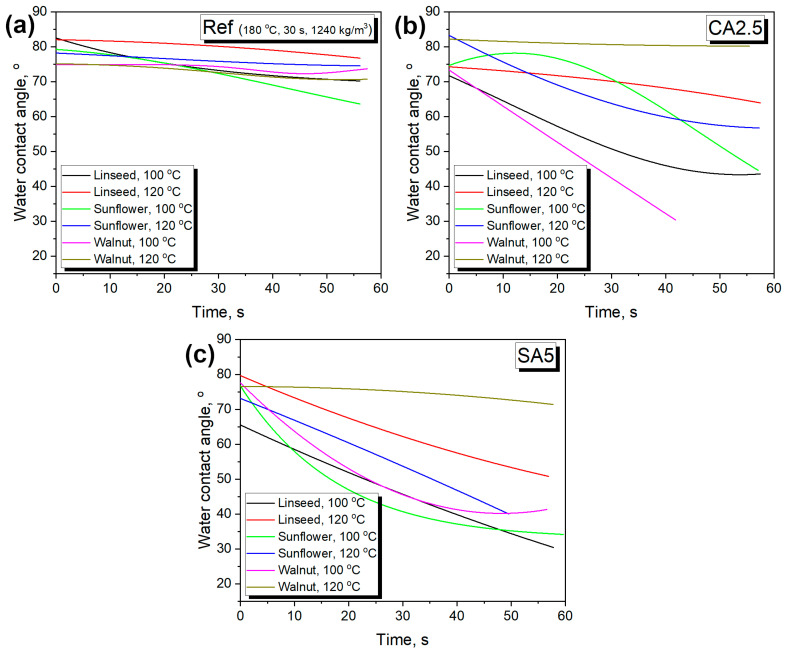
The impact of applied oil coating on variations in water contact angle over time for (**a**) reference sample—15; (**b**) sample containing 2.5 wt% of CA; and (**c**) sample containing 5.0 wt% of SA.

Based on their mechanical properties, for the analysis of the oil coating procedure, samples 15 (reference without organic acid modification), 21 (2.5 wt% CA addition), and 24 (5.0 wt% SA addition) were selected. They were coated with linseed, sunflower, and walnut oils and subjected to thermooxidation at 100 and 120 °C. Figure 13 presents the impact of the applied oil coating on WCA variations over time. It is clear that, compared to the uncoated samples (Figure 8), the rate of WCA decrease was substantially reduced, indicating the solution’s efficiency. Nevertheless, the impact of the oil coating varied significantly among the analyzed samples, which may have been associated with structural features resulting from formulation changes. As shown in Figure 4, the introduction of organic acids as modifiers increased the porosity, facilitating the penetration of the material when oil was applied as a coating and limiting the formation of a protective surface layer. Such an effect was confirmed by the reduced WCA decrease after thermooxidation at 120 °C compared to the lower temperature. Faster oxidation enabled more efficient formation of the protective hydrophobic layer. A similar effect was noted for the reference material (sample 15); however, lower porosity minimized the temperature impact.

The increased hydrophobicity of the surface of products coated with oils and subjected to oxidation processes, leading to crosslinking, may have resulted from two simultaneous phenomena: (i) the intensity of the oil oxidation process and (ii) the initial oil viscosity and viscosity increase during aging, which affected the wetting of the product structure and development of a continuous hydrophobic layer. Among the oils used, linseed oil showed the fastest decay of the thermal phenomena observed in the heating and cooling curves, with a viscosity comparable to that of walnut oil. The observed changes in the highest effectiveness in maintaining the high WCA of products coated with 120 °C-oxidized linseed and walnut oils were therefore related to both their lowest viscosity at the beginning of the coating process, which resulted in good wetting of the product surface, and their high degree of oxidation, as inferred from the summarized FTIR (Figure 11) and calorimetric test results (Figure 12). The recommended oil application procedure involved using the highest temperature (120 °C) and applying linseed oil for products with unmodified BSG and walnut oil for compositions that use citric and succinic acid to support the solidification process.

### 3.6. Manufacturing of the Final Product—Disposable Plates

**Figure 14:**
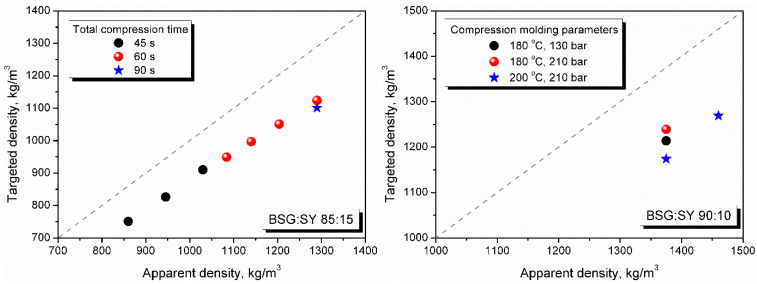
The impact of input mass on the apparent density of products developed with varying procedures (total compression time, temperature, and pressure) for materials prepared with BSG/SY ratios of (**left**) 85:15 and (**right**) 90:10. The dotted line indicates the equal targeted and apparent density yielding compression ratio of 1.

The manufacturing of disposable plates was aimed at verifying the industrial potential of the developed materials. Considering the noticeable changes in the final product’s shape compared to rectangular samples, proper adjustments to the processing parameters were necessary, as presented in Table 3, which provides the processing parameters for plate manufacturing. Precise filling of the mold cavity with non-flowing input material required a significant increase in pressure, from 20 bar to 130 bar. Such a change also required adjustments to the processing time and procedure, which for plates included a degassing phase. Initially, the impact of input mass on the apparent density of products produced under varying procedures (total compression time, temperature, and pressure) was assessed (Figure 14). It can be seen that the higher pressure, compared to 20–30 bar applied during the laboratory tests, significantly reduced the final product’s porosity, achieving a higher targeted density. In the case of laboratory tests, samples with a targeted density of 1240 kg/m^3^ showed a compression ratio of 0.66–0.76, whereas for the produced disposable plates, values ranged from 0.85 to 0.90.

On the other hand, unfavorable effects were also noted. For a BSG/SY ratio of 85:15, elongation of the total compression time for the applied manufacturing procedure (compression for 1 s → degassing for 2 s → compression for 30 s → degassing for 2 s → second compression for 15/30/60 s) adversely affected the product’s porosity. Such an effect could be associated with excessive degradation and the generation of volatiles, which, under high pressure, were unable to escape the mold cavity and induced porosity in the plates. Accordingly, increasing the compression temperature from 180 to 200 °C for a BSG/SY ratio of 90:10 reduced the compression ratio despite the introduction of a third degassing phase.

**Figure 15:**
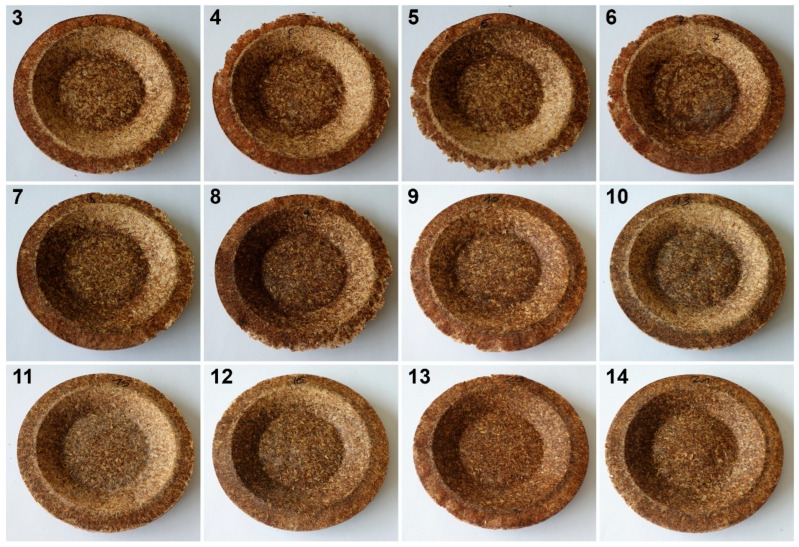
The appearance of disposable plates manufactured with different formulations and processing parameters. Numbers refer to the formulations provided in Table 3.

In addition to the porosity analysis, visual inspection (Figure 15) revealed inaccuracies in mold filling that affected the appearance of the developed plates. For the lower values of targeted density, ranging from 860 to 1140 kg/m^3^ (samples 3–6), significant irregularities on plate edges could be observed, indicating inadequate mass input. For samples 7–9, with a targeted density of 1200–1290 kg/m^3^, the filling precision was noticeably higher, but the products were still hardly flawless.

Nevertheless, proper adjustments to the formulation (shifting the BSG/SY ratio from 85:15 to 90:10) and exceeding a density of 1375 kg/m^3^ enabled precise filling of the mold cavity and the manufacturing of disposable plates without drawbacks (samples 10–14). Comparing samples 9 and 10 (increased BSG share), a more accurate representation of the plate’s shape is observed, with fewer internal voids resulting from inefficient moisture evaporation during the degassing phase. Introducing an additional degassing phase for sample 11 (3 degassing phases with the same compression time) further improved the plate’s cohesion, similar to the pressure increase for sample 12 (from 130 to 210 bar). Subsequent temperature increase (samples 13 and 14) and exceeding a density of 1450 kg/m^3^ (sample 14) led to the most favorable effects, highlighting the most efficient moisture evaporation, as well as browning of the surface related to partial thermal degradation of brewery by-products, including strongly desired NEBRs [94].

**Figure 16:**
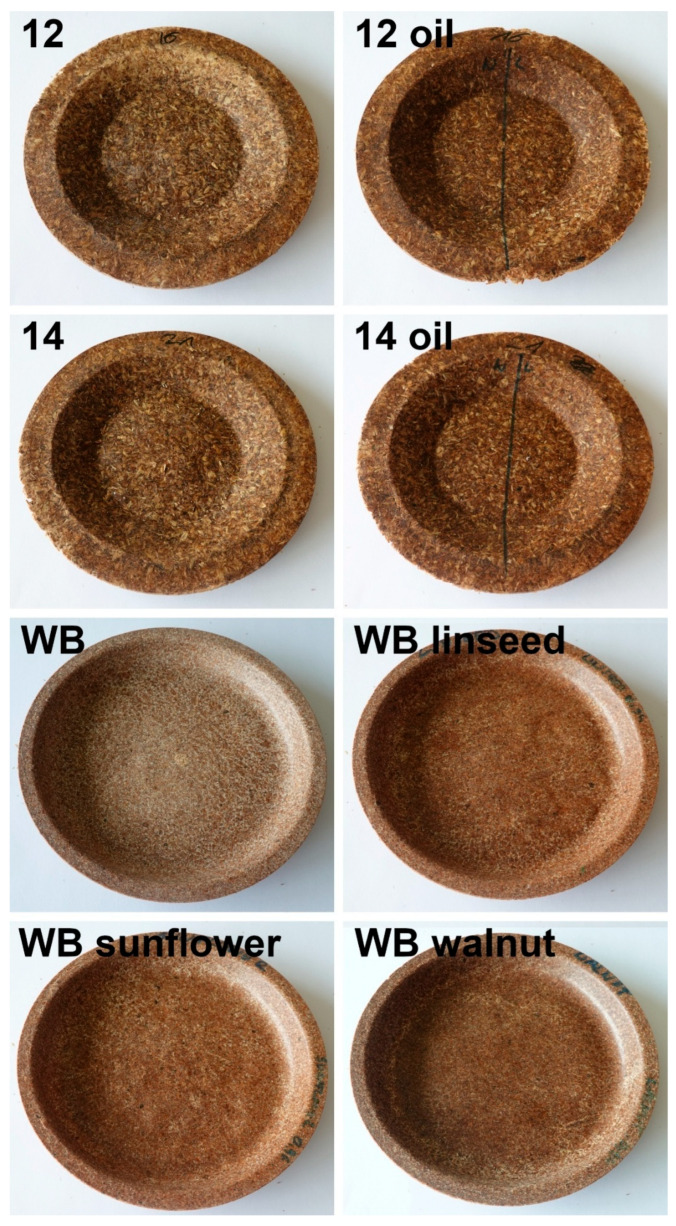
The appearance of selected plate samples before and after coating with vegetable oils. Numbers refer to the formulations provided in Table 3.

Selected samples (12 and 14) were coated with oil to evaluate the impact on appearance and potential plate swelling. The appearance of the analyzed samples is presented in Figure 16. Coating the plates did not affect their structure. No pores were generated, which, together with the darkening of their surface (in line with changes in oil color during thermooxidation), indicated the efficient formation of a protective layer.

A similar test was performed on commercially available plates manufactured from wheat bran. Accordingly, surface darkening was noted, which could be attributed to the thermooxidation of oils used as coatings. Nevertheless, unlike the BSG/SY plates, significant absorption of oil was observed in the wheat bran plates, resulting in void formation and stratification of the plate structure. Such an effect could be induced by the lower particle size of wheat bran compared to that of BSG used in the present study. Moreover, the presence of protein-rich SY significantly enhanced NEBRs and boosted the generation of melanoidins (the extent of which could be indirectly assessed by the browning level [95,96]), which may have affected surface wettability and limited the absorption of oil into the structure.

### 3.7. Limitations and Further Considerations for Potential Applications

Despite auspicious results related to the mechanical performance and moisture resistance of the developed BSG/SY materials and disposable plates, there are limitations regarding the implementation of the presented solution. First, economic profitability must be comprehensively assessed, taking into account the price of raw materials and the energy inputs associated with the particular manufacturing processes. Next, a proper analysis related to the approval of material for food contact use has to be conducted. The migration of compounds from the developed materials into food products or liquids should be comprehensively assessed. In further work, this aspect could be addressed, e.g., by Soxhlet extraction, solid-phase extraction, and headspace analysis using specialized stationary emission chambers for thermal extraction of volatile organic compounds emission [97,98]. Based on the gathered information, the potential health impacts and allergenicity could be assessed. Except for the compounds originating from applied raw materials, the impact of non-intentionally added substances should be assessed, as this is a major concern for plant fiber-based food contact materials, as recently described by Tang et al. [99]. Nevertheless, unlike plastic-based materials, which may release significant amounts of microplastics and residual additives and processing aids, the absence of added plastics in the presented case is beneficial. Therefore, the only non-intentionally added substances can be attributed to contamination of the applied raw materials or to changes in their chemical composition induced by high-temperature and high-pressure processing. Fortunately, beer production is considered a relatively clean process, as it is highly sensitive to microbial contamination [100,101], thereby benefiting the purity of its by-products. Numerous studies on the use of BSG [102,103,104,105,106] and SY [107,108,109,110] as raw materials in food products confirm their safety for food contact applications, especially considering similar processing temperatures. Moreover, BSG has already been implemented in the commercial manufacturing of food products in various countries worldwide [104]. Among the available BSG-based food products are flour, bread, pizza, pasta, baking mixes, sweet snacks, crackers, cereals, and seasonings [104]. Other than BSG and SY, the proposed solution uses various vegetable oils, which are also commonly used in the food sector, and organic acids, which are used as food additives. Although we acknowledge the need for a detailed analysis of their behavior during the processing proposed in the study, they are already approved for use in food products, which allows us to look positively at their potential use in the manufacturing of disposable products.

## 4. Conclusions

This study presents a novel approach to manufacturing disposable products from BSG by introducing SY as a potential binder of brewery wastes. It is very innovative, as waste yeast has rarely been reported for binder applications, especially in its direct form without additional modifications. A proper combination of BSG and SY in an 85:15 ratio, similar to that used to quantify waste generation, enabled the manufacture of materials characterized with a flexural strength exceeding 5 MPa, a flexural modulus exceeding 1 GPa, and a hardness exceeding 50 ShD. Moreover, these values could be enhanced by shifting the BSG/SY ratio toward a higher BSG share. Introducing common organic acids as sustainable crosslinking agents further increased the flexural strength and hardness. Due to the nature of the applied raw materials and their inherent moisture susceptibility, the materials exhibited high surface wettability and low water contact angles. Given their potential applications, the final products were coated with vegetable oils, which, upon thermooxidation-induced crosslinking, formed a protective layer on the material’s surface. Detailed analysis of the crosslinking process enabled the selection of appropriate oils, the application of which provided protection against moisture and humidity. Oil application significantly increased the water contact angle (by over 40°) and limited water penetration into the material’s structure, potentially broadening the application range.

Finally, the application potential was confirmed through the efficient manufacturing of disposable plates, which can be potentially implemented in industrial practice after detailed economic calculations and proper assessment of market demand. Notably, the study’s results address not only issues in the beer sector but also a key demand of the plastic industry: the manufacture of disposable products. The proposed solution fully aligns with this concept by keeping brewery waste in a loop, providing a step toward a circular economy. Due to the simplicity of the reported manufacturing process and the geographical distribution of breweries that generate applied waste, it can be implemented with minimal location constraints, revitalizing local economies. Nevertheless, potential applications should meet local requirements related to food contact materials, including extractables and their migration, odor, and the stability of the developed materials and coatings. Therefore, proper food compliance analysis is required, which will be the topic of further studies.

## Figures and Tables

**Table 1:** Literature-based composition data of applied vegetable oils.

Oil	Saturated Fatty Acids	Monounsaturated Fatty Acids	Polyunsaturated Fatty Acids	Acid Value, mg KOH/g	Peroxide Value, meq O_2_/kg Oil	Refs.	Viscosity, mPa·s
Hazelnut	7.0–15.6	77.8–87.1	1.9–15.1	0.14–3.26	0.28–12.50	[30,31,32,33]	49.0 ± 0.3
Linseed	5.7–11.1	11.2–23.7	65.3–81.8	0.53–3.15	1.23–4.50	[34,35]	30.8 ± 0.3
Peanut	20.9–22.0	41.7–44.8	34.3–36.2	0.09–2.19	0.33–7.72	[36,37,38]	51.6 ± 0.4
Sesame	14.7–18.2	35.5–41.6	40.8–49.1	0.75–3.68	0.92–8.99	[39,40,41]	42.3 ± 0.3
Sunflower	9.6–10.1	20.7–26.5	62.8–69.6	0.24–4.10	1.03–5.38	[42,43,44,45]	39.5 ± 0.3
Walnut	4.1–13.6	12.8–25.4	61.8–77.7	0.05–0.58	1.12–3.10	[46,47,48]	34.6 ± 0.3

**Table 2:** Formulations and processing parameters applied during the manufacturing of BSG/SY materials. Contents of particular components refer to the applied forms: BSG—dried, 10.0 wt% moisture content; SY—as-received, no drying, 83.5 wt% moisture content; CA, SA, and TA—as-received, no drying, 0.19, 0.08, and 0.04 wt% moisture content, respectively.

Sample	Content, wt%					Initial Moisture Content, wt%	Water to Dry Solids Ratio	Targeted Density, kg/m^3^	Temperature, °C	Pressure, bar	Time, s
	BSG	SY	CA	SA	TA						
1	75	25	-	-	-	28.38	1:2.52	620	200	20	10
2	75	25	-	-	-	28.38	1:2.52	620	200	20	20
3	75	25	-	-	-	28.38	1:2.52	620	200	20	30
4	75	25	-	-	-	28.38	1:2.52	620	200	20	60
5	75	25	-	-	-	28.38	1:2.52	620	200	20	120
6	75	25	-	-	-	28.38	1:2.52	620	200	20	180
7	75	25	-	-	-	28.38	1:2.52	723	200	20	120
8	75	25	-	-	-	28.38	1:2.52	826	200	20	120
9	75	25	-	-	-	28.38	1:2.52	930	200	20	120
10	85	15	-	-	-	21.03	1:3.76	930	180	20	30
11	85	15	-	-	-	21.03	1:3.76	930	200	20	30
12	85	15	-	-	-	21.03	1:3.76	930	220	20	30
13	85	15	-	-	-	21.03	1:3.76	930	200	25	30
14	85	15	-	-	-	21.03	1:3.76	930	200	30	30
15	85	15	-	-	-	21.03	1:3.76	1240	180	20	30
16	85	15	-	-	-	21.03	1:3.76	1240	200	20	30
17	85	15	-	-	-	21.03	1:3.76	1240	220	20	30
18	92.5	7.5	-	-	-	15.51	1:5.45	1240	200	20	30
19	92.5	7.5	-	-	-	15.51	1:5.45	1240	200	25	30
20	92.5	7.5	-	-	-	15.51	1:5.45	1240	200	30	30
21	82.87	14.63	2.5	-	-	20.51	1:3.88	1240	180	20	30
22	80.75	14.25	5.0	-	-	19.98	1:4.00	1240	180	20	30
23	76.50	13.50	10.0	-	-	18.94	1:4.28	1240	180	20	30
24	80.75	14.25	-	5.0	-	19.98	1:4.01	1240	180	20	30
25	80.75	14.25	-	-	5.0	19.98	1:4.01	1240	180	20	30

**Table 3:** Formulations and processing parameters applied during the manufacturing of BSG/SY disposable plates. Abbreviation DG indicates the degassing phase lasting 2 s. Contents of particular components refer to the applied forms: BSG—dried, 10.0 wt% moisture content; SY—as-received, 83.5 wt% moisture content.

Sample	Content, wt%		Initial Moisture Content, wt%	Water to Dry Solids Ratio	Targeted Density, kg/m^3^	Temperature, °C	Pressure, bar	Procedure
	BSG	SY						
1	85	15	21.03	1:3.76	860	180	130	1 s → DG → 30 s → DG → 15 s (total 50 s)
2	85	15	21.03	1:3.76	945	180	130	1 s → (DG) → 30 s → DG → 15 s (50 s)
3	85	15	21.03	1:3.76	1030	180	130	1 s → (DG) → 30 s → DG → 15 s (50 s)
4	85	15	21.03	1:3.76	1030	180	130	1 s → DG → 30 s → DG → 30 s (65 s)
5	85	15	21.03	1:3.76	1084	180	130	1 s → DG → 30 s → DG → 30 s (65 s)
6	85	15	21.03	1:3.76	1140	180	130	1 s → DG → 30 s → DG → 30 s (65 s)
7	85	15	21.03	1:3.76	1204	180	130	1 s → DG → 30 s → DG → 30 s (65 s)
8	85	15	21.03	1:3.76	1290	180	130	1 s → DG → 30 s → DG → 30 s (65 s)
9	85	15	21.03	1:3.76	1290	180	130	1 s → DG → 30 s → DG → 60 s (95 s)
10	90	10	17.35	1:4.76	1290	180	130	1 s → DG → 30 s → DG → 60 s (95 s)
11	90	10	17.35	1:4.76	1375	180	130	1 s → DG → 30 s → DG → 30 s → DG → 30 s (97 s)
12	90	10	17.35	1:4.76	1375	180	210	1 s → DG → 30 s → DG → 30 s → DG → 30 s (97 s)
13	90	10	17.35	1:4.76	1375	200	210	1 s → DG → 30 s → DG → 30 s → DG → 30 s (97 s)
14	90	10	17.35	1:4.76	1460	200	210	1 s → DG → 30 s → DG → 30 s → DG → 30 s (97 s)

**Table 4:** Initial appearance of oil samples and changes resulting from thermooxidation at 120 °C.

Oil	Aging Time, Days			
	0	3	7	14
Hazelnut	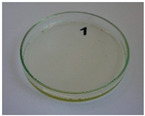	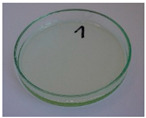	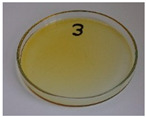	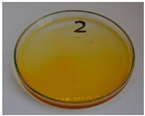
Linseed	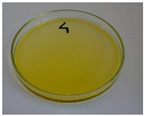	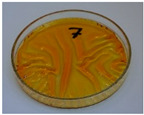	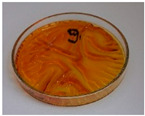	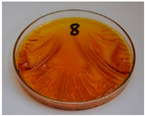
Peanut	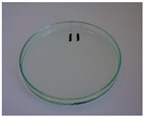	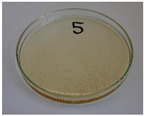	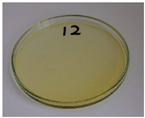	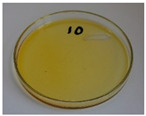
Sesame	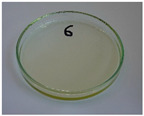	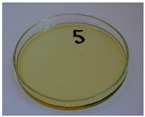	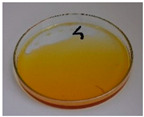	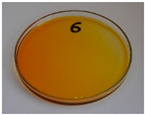
Sunflower	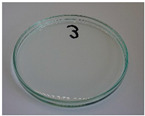	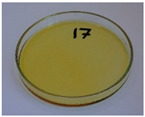	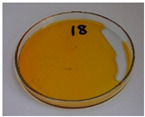	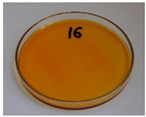
Walnut	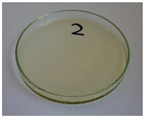	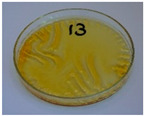	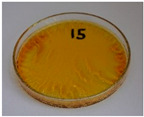	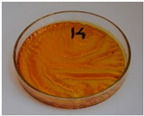

**Table 5:** Parameters quantifying the initial color of applied oils and their changes after thermooxidation at different temperatures.

Oil	Aging Time, Days	Color Parameters					
		L*	a*	b*	Chroma	Hue, °	BI
Hazelnut	0	82.29 ± 0.62	−0.23 ± 0.11	0.59 ± 0.29	0.64 ± 0.30	65.88 ± 11.24	−0.13 ± 0.08
Linseed		74.98 ± 1.75	−0.02 ± 0.31	5.07 ± 1.36	5.08 ± 1.37	87.39 ± 1.09	0.65 ± 0.28
Peanut		82.84 ± 0.15	0.58 ± 0.02	−1.94 ± 0.05	2.03 ± 0.05	73.29 ± 0.70	0.28 ± 0.02
Sesame		75.58 ± 2.48	0.28 ± 0.08	0.66 ± 0.20	0.72 ± 0.19	65.65 ± 8.32	0.36 ± 0.09
Sunflower		81.83 ± 0.90	0.36 ± 0.08	−1.19 ± 0.27	1.25 ± 0.28	72.93 ± 3.43	0.18 ± 0.06
Walnut		77.52 ± 2.30	0.30 ± 0.08	0.21 ± 0.49	0.55 ± 0.22	52.38 ± 12.51	0.31 ± 0.08
Treatment at 120 °C							
Hazelnut	3	82.09 ± 0.43	0.56 ± 0.01	−1.75 ± 0.06	1.83 ± 0.05	72.13 ± 0.79	0.29 ± 0.02
	7	70.39 ± 0.90	−1.27 ± 0.11	39.08 ± 2.96	39.10 ± 2.96	88.12 ± 0.26	4.25 ± 0.52
	14	64.10 ± 1.27	7.81 ± 1.41	59.45 ± 5.05	59.96 ± 5.18	82.56 ± 0.79	18.30 ± 2.25
Linseed	3	63.29 ± 2.20	9.36 ± 0.73	59.63 ± 4.43	60.36 ± 4.46	81.08 ± 0.43	20.28 ± 1.05
	7	62.03 ± 2.08	21.77 ± 3.48	58.43 ± 6.02	62.45 ± 5.90	69.47 ± 3.37	33.94 ± 3.43
	14	46.16 ± 4.62	27.65 ± 5.40	58.18 ± 3.73	64.64 ± 3.26	64.59 ± 5.05	53.52 ± 8.13
Peanut	3	82.45 ± 2.29	0.41 ± 0.17	1.44 ± 0.46	1.51 ± 0.45	72.89 ± 7.48	0.53 ± 0.15
	7	72.80 ± 3.91	−1.36 ± 0.45	14.15 ± 3.42	14.23 ± 3.40	84.20 ± 2.49	0.58 ± 0.71
	14	70.31 ± 0.65	0.09 ± 0.79	50.16 ± 4.05	50.17 ± 4.05	89.20 ± 0.38	7.33 ± 1.29
Sesame	3	82.33 ± 0.52	0.40 ± 0.19	−0.50 ± 0.17	0.66 ± 0.20	54.21 ± 17.55	0.29 ± 0.16
	7	66.43 ± 0.55	9.30 ± 0.51	70.30 ± 3.31	70.92 ± 3.35	82.47 ± 0.08	21.04 ± 1.27
	14	65.63 ± 1.55	14.37 ± 4.36	82.80 ± 3.73	84.11 ± 4.42	80.27 ± 2.48	28.93 ± 5.91
Sunflower	3	72.75 ± 0.24	−1.41 ± 0.15	39.28 ± 0.65	39.30 ± 0.66	87.95 ± 0.20	4.00 ± 0.10
	7	65.81 ± 1.61	9.96 ± 1.32	73.19 ± 2.79	73.88 ± 2.72	82.23 ± 1.14	22.47 ± 1.91
	14	62.38 ± 0.77	21.56 ± 1.38	85.60 ± 1.63	88.28 ± 1.73	75.86 ± 0.80	38.68 ± 1.93
Walnut	3	65.56 ± 2.04	1.46 ± 0.37	50.41 ± 3.62	50.43 ± 3.63	88.35 ± 0.33	9.43 ± 0.68
	7	62.61 ± 0.64	12.67 ± 0.47	51.37 ± 1.41	52.91 ± 1.35	76.13 ± 0.68	22.73 ± 0.66
	14	53.19 ± 1.67	19.21 ± 4.02	56.11 ± 4.01	59.41 ± 4.42	71.17 ± 3.56	35.97 ± 5.80
Treatment at 100 °C							
Linseed	3	75.99 ± 3.96	−0.89 ± 2.60	48.17 ± 4.71	48.24 ± 4.69	86.95 ± 0.98	5.46 ± 2.33
	7	66.20 ± 5.27	5.34 ± 3.83	51.82 ± 6.78	52.18 ± 7.06	84.36 ± 3.54	14.00 ± 5.79
	14	63.54 ± 4.59	11.22 ± 2.86	56.13 ± 7.27	57.28 ± 7.50	78.74 ± 2.21	21.69 ± 4.03
Sunflower	3	75.27 ± 1.49	−0.46 ± 0.14	1.60 ± 1.04	1.68 ± 1.02	68.01 ± 14.52	−0.22 ± 0.03
	7	78.05 ± 1.19	−2.73 ± 0.31	33.18 ± 3.64	33.30 ± 3.60	85.19 ± 1.09	1.68 ± 0.76
	14	75.00 ± 0.76	−1.59 ± 0.34	49.87 ± 2.89	49.90 ± 2.87	88.15 ± 0.53	5.16 ± 0.78
Walnut	3	86.96 ± 7.18	−2.49 ± 0.62	19.64 ± 6.34	19.80 ± 6.36	82.51 ± 1.31	0.13 ± 0.45
	7	71.26 ± 2.62	−0.65 ± 0.85	51.12 ± 4.23	51.14 ± 4.22	89.01 ± 0.77	6.58 ± 1.34
	14	69.55 ± 2.60	4.26 ± 2.03	58.88 ± 3.49	59.06 ± 3.48	85.85 ± 2.01	13.14 ± 2.46
Treatment at 80 °C							
Linseed	3	78.05 ± 1.56	−3.00 ± 1.51	41.25 ± 4.04	41.38 ± 4.06	85.84 ± 2.00	2.46 ± 1.46
	7	67.76 ± 4.16	0.19 ± 1.62	42.66 ± 3.03	42.69 ± 3.04	88.19 ± 1.01	6.66 ± 2.33
	14	66.07 ± 3.25	2.63 ± 1.23	46.86 ± 5.56	46.94 ± 5.59	86.86 ± 1.35	10.10 ± 2.05
Sunflower	3	75.10 ± 1.44	−0.08 ± 0.11	−1.53 ± 0.34	1.54 ± 0.34	85.01 ± 3.40	−0.26 ± 0.10
	7	79.94 ± 0.55	−0.82 ± 0.09	7.41 ± 0.43	7.45 ± 0.44	83.74 ± 0.41	0.17 ± 0.04
	14	76.98 ± 0.78	−3.52 ± 0.32	31.50 ± 1.70	31.70 ± 1.66	83.58 ± 0.91	0.72 ± 0.53
Walnut	3	74.64 ± 0.94	−0.29 ± 0.14	2.23 ± 0.69	2.25 ± 0.70	82.94 ± 2.14	0.01 ± 0.06
	7	76.94 ± 3.60	−3.55 ± 0.75	30.36 ± 3.30	30.57 ± 3.31	83.33 ± 1.33	0.56 ± 0.69
	14	73.87 ± 1.88	−1.77 ± 0.44	38.23 ± 3.74	38.27 ± 3.74	87.35 ± 0.62	3.43 ± 0.56

**Table 6:** Initial appearance of linseed, sunflower, and walnut oil samples and changes resulting from thermooxidation at 80 and 100 °C.

	Thermal treatment at 80 °C			
Oil	Aging time, days			
	0	3	7	14
Linseed	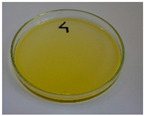	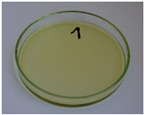	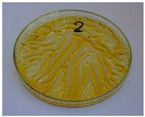	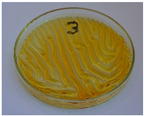
Sunflower	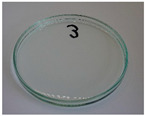	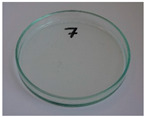	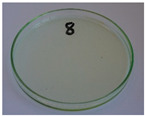	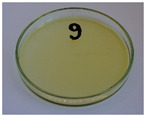
Walnut	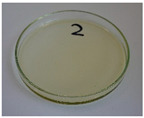	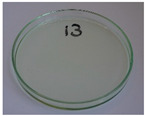	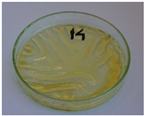	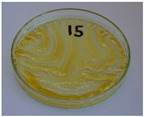
	Thermal treatment at 100 °C			
Oil	Aging time, days			
	0	3	7	14
Linseed	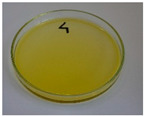	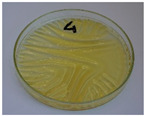	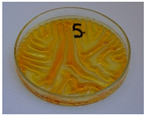	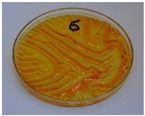
Sunflower	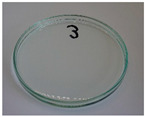	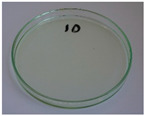	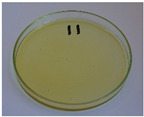	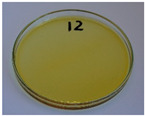
Walnut	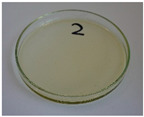	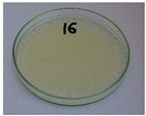	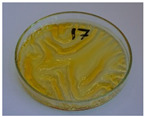	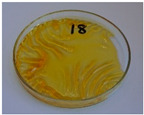

## Data Availability

Data are available at https://doi.org/10.5281/zenodo.17603758.

## Supplementary Materials

The following supporting information can be downloaded at: https://www.mdpi.com/article/10.3390/foods15050860/s1, Figure S1. FTIR spectra of hazelnut oil subjected to thermooxidation at 120 °C; Figure S2. FTIR spectra of peanut oil subjected to thermooxidation at 120 °C; Figure S3. FTIR spectra of sesame oil subjected to thermooxidation at 120 °C; Figure S4. FTIR spectra of linseed oil subjected to thermooxidation at 80 °C; Figure S5. FTIR spectra of linseed oil subjected to thermooxidation at 100 °C; Figure S6. FTIR spectra of sunflower oil subjected to thermooxidation at 80 °C; Figure S7. FTIR spectra of sunflower oil subjected to thermooxidation at 100 °C; Figure S8. FTIR spectra of sunflower oil subjected to thermooxidation at 120 °C; Figure S9. FTIR spectra of walnut oil subjected to thermooxidation at 80 °C; Figure S10. FTIR spectra of walnut oil subjected to thermooxidation at 100 °C; Figure S11. FTIR spectra of walnut oil subjected to thermooxidation at 120 °C; Figure S12. DSC heating and cooling curves of hazelnut oil subjected to 120 °C at 0, 3, 7, and 14 days; Figure S13. DSC heating and cooling curves of linseed oil subjected to 80 °C at 0, 3, 7, and 14 days; Figure S14. DSC heating and cooling curves of linseed oil subjected to 100 °C at 0, 3, 7, and 14 days; Figure S15. DSC heating and cooling curves of linseed oil subjected to 120 °C at 0, 3, 7, and 14 days; Figure S16. DSC heating and cooling curves of peanut oil subjected to 120 °C at 0, 3, 7, and 14 days; Figure S17. DSC heating and cooling curves of sesame oil subjected to 120 °C at 0, 3, 7, and 14 days; Figure S18. DSC heating and cooling curves of sunflower oil subjected to 80 °C at 0, 3, 7, and 14 days; Figure S19. DSC heating and cooling curves of sesame oil subjected to 100 °C at 0, 3, 7, and 14 days; Figure S20. DSC heating and cooling curves of sunflower oil subjected to 120 °C at 0, 3, 7, and 14 days; Figure S21. DSC heating and cooling curves of walnut oil subjected to 80 °C at 0, 3, 7, and 14 days; Figure S22. DSC heating and cooling curves of walnut oil subjected to 100 °C at 0, 3, 7, and 14 days; Figure S23. DSC heating and cooling curves of walnut oil subjected to 120 °C at 0, 3, 7, and 14 days; Table S1. Dry-basis formulations and processing parameters applied during the manufacturing of BSG/SY materials; Table S2. Dry-basis formulations and processing parameters applied during the manufacturing of BSG/SY disposable plates; Table S3. Thermal parameters obtained from DSC for edible oils before and after 3, 7, and 14-day exposure to elevated temperature (80, 100, and 120 °C) in oxidative atmosphere; TM—melting temperature; TC—crystallization temperature.

## Abbreviations

The following abbreviations are used in this manuscript:

ATR	Attenuated total reflectance
BSG	Brewers’ spent grain
CA	Citric acid
DG	Degassing
DSC	Differential scanning calorimetry
EU	European Union
FTIR	Fourier transform infrared spectroscopy
NEBRs	Non-enzymatic browning reactions
SA	Succinic acid
SY	Spent yeast
TA	L-(+)-tartaric acid
TAG	Triacyloglycerol
TGA	Thermogravimetric analysis
WCA	Water contact angle
