# Supplementary material for: Turning Disposed into Disposable—Development of Single-Use Products from Underutilized Brewery Wastes
Source: Foods. 2026 Mar 4;15(5):860. doi: 10.3390/foods15050860 (PMC12985305; doi:10.3390/foods15050860)
Supplement: Supplementary file 1 [file foods-15-00860-s001.zip › foods-4148274-supplementary.pdf]

foods

Article

Turning Disposed into Disposable—Development of Single-Use Products from Underutilized Brewery Wastes

Aleksander Hejna * and Mateusz Barczewski

Institute of Material Technology, Poznan University of Technology, Piotrowo 3, 61-138 Poznań, Poland; mateusz.barczewski@put.poznan.pl
* Correspondence: aleksander.hejna@put.poznan.pl

S1. Detailed sample manufacturing information

Table S1. Dry-basis formulations and processing parameters applied during the manufacturing of BSG/SY materials.

Sample	Content, wt%					Targeted density, kg/m^3	Temperature, °C	Pressure, bar	Time, s
	BSG	SY	CA	SA	TA				
1	94.24	5.76	-	-	-	620	200	20	10
2	94.24	5.76	-	-	-	620	200	20	20
3	94.24	5.76	-	-	-	620	200	20	30
4	94.24	5.76	-	-	-	620	200	20	60
5	94.24	5.76	-	-	-	620	200	20	120
6	94.24	5.76	-	-	-	620	200	20	180
7	94.24	5.76	-	-	-	723	200	20	120
8	94.24	5.76	-	-	-	826	200	20	120
9	94.24	5.76	-	-	-	930	200	20	120
10	96.87	3.13	-	-	-	930	180	20	30
11	96.87	3.13	-	-	-	930	200	20	30
12	96.87	3.13	-	-	-	930	220	20	30
13	96.87	3.13	-	-	-	930	200	25	30
14	96.87	3.13	-	-	-	930	200	30	30
15	96.87	3.13	-	-	-	1240	180	20	30
16	96.87	3.13	-	-	-	1240	200	20	30
17	96.87	3.13	-	-	-	1240	220	20	30
18	98.54	1.46	-	-	-	1240	200	20	30
19	98.54	1.46	-	-	-	1240	200	25	30
20	98.54	1.46	-	-	-	1240	200	30	30
21	93.82	3.04	3.14	-	-	1240	180	20	30
22	90.82	2.94	6.24	-	-	1240	180	20	30
23	84.94	2.75	12.31	-	-	1240	180	20	30
24	90.82	2.94	-	6.24	-	1240	180	20	30
25	90.82	2.94	-	-	6.24	1240	180	20	30

Table S2. Dry-basis formulations and processing parameters applied during the manufacturing of BSG/SY disposable plates.

Sample	Content, wt%		Targeted density, kg/m³	Temperature, °C	Pressure, bar	Procedure
	BSG	SY				
1	96.87	3.13	860	180	130	1 s→DG→30 s→DG→15 s (total 50 s)
2	96.87	3.13	945	180	130	1 s→(DG)→30 s→DG→15 s (50 s)
3	96.87	3.13	1030	180	130	1 s→(DG)→30 s→DG→15 s (50 s)
4	96.87	3.13	1030	180	130	1 s→DG→30 s→DG→30 s (65 s)
5	96.87	3.13	1084	180	130	1 s→DG→30 s→DG→30 s (65 s)
6	96.87	3.13	1140	180	130	1 s→DG→30 s→DG→30 s (65 s)
7	96.87	3.13	1204	180	130	1 s→DG→30 s→DG→30 s (65 s)
8	96.87	3.13	1290	180	130	1 s→DG→30 s→DG→30 s (65 s)
9	96.87	3.13	1290	180	130	1 s→DG→30 s→DG→60 s (95 s)
10	98.00	2.00	1290	180	130	1 s→DG→30 s→DG→60 s (95 s)
11	98.00	2.00	1375	180	130	1 s→DG→30 s→DG→30 s→DG→30 s (97 s)
12	98.00	2.00	1375	180	210	1 s→DG→30 s→DG→30 s→DG→30 s (97 s)
13	98.00	2.00	1375	200	210	1 s→DG→30 s→DG→30 s→DG→30 s (97 s)
14	98.00	2.00	1460	200	210	1 s→DG→30 s→DG→30 s→DG→30 s (97 s)

S2. Additional results from differential scanning calorimetry

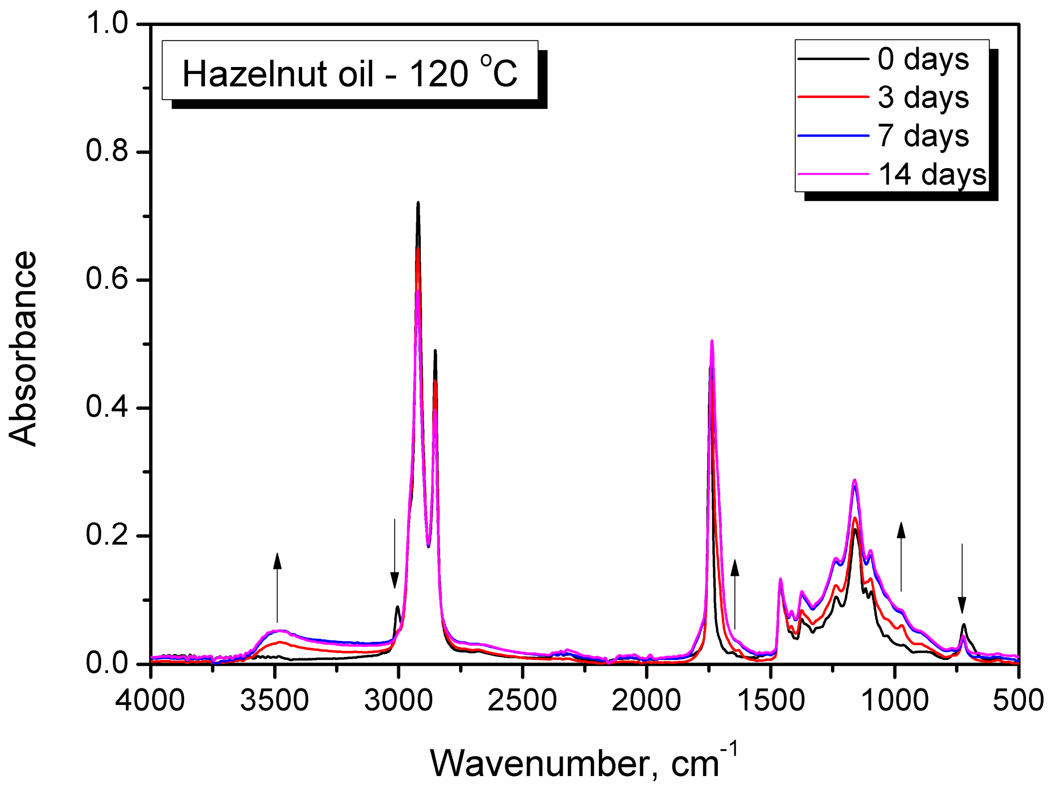

Figure S1. FTIR spectra of hazelnut oil subjected to thermooxidation at 120 °C.

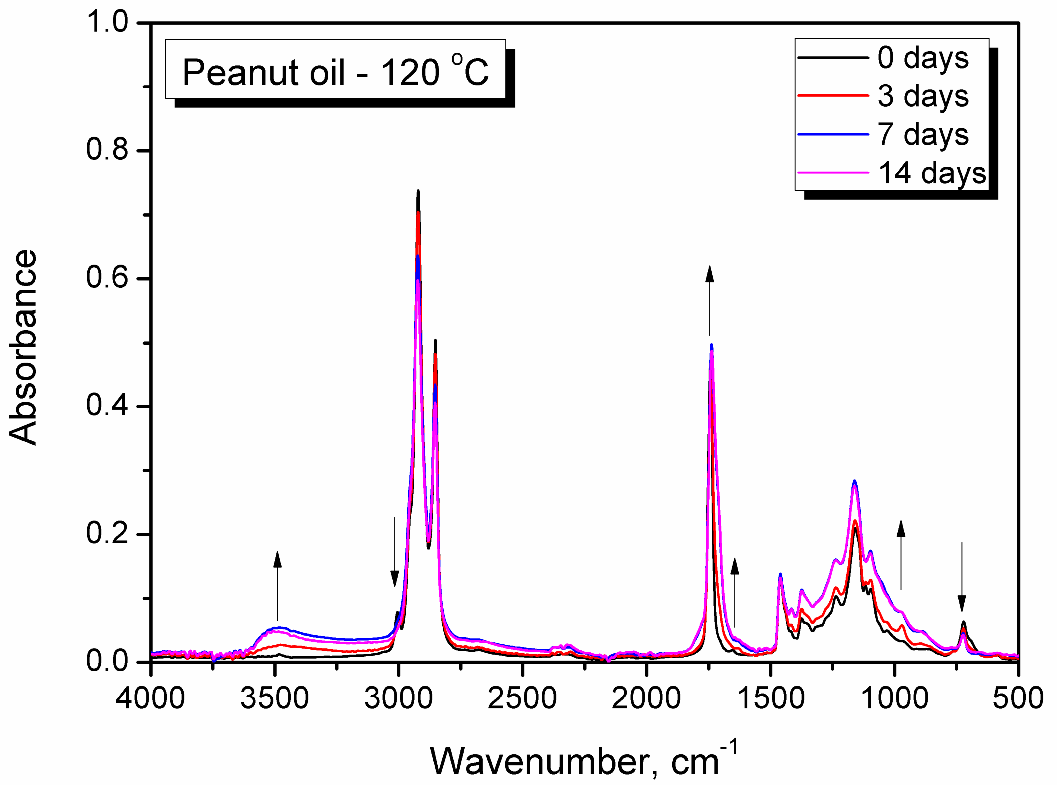

Figure S2. FTIR spectra of peanut oil subjected to thermooxidation at 120 °C.

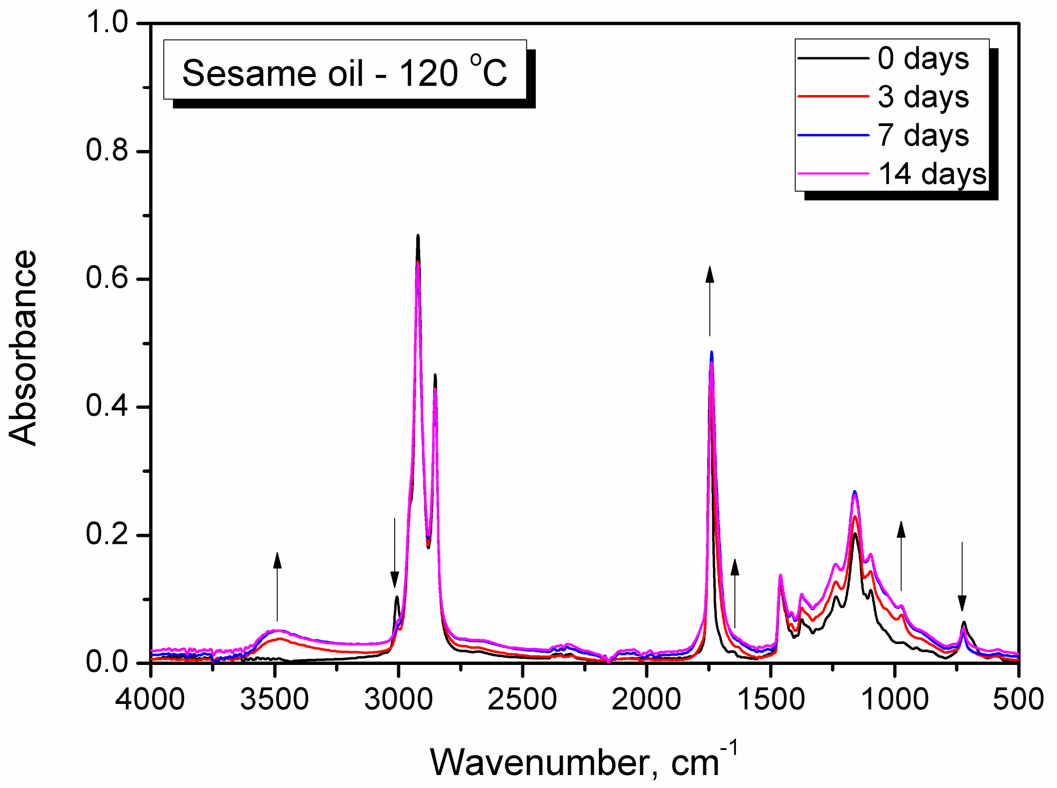

Figure S3. FTIR spectra of sesame oil subjected to thermooxidation at 120 °C.

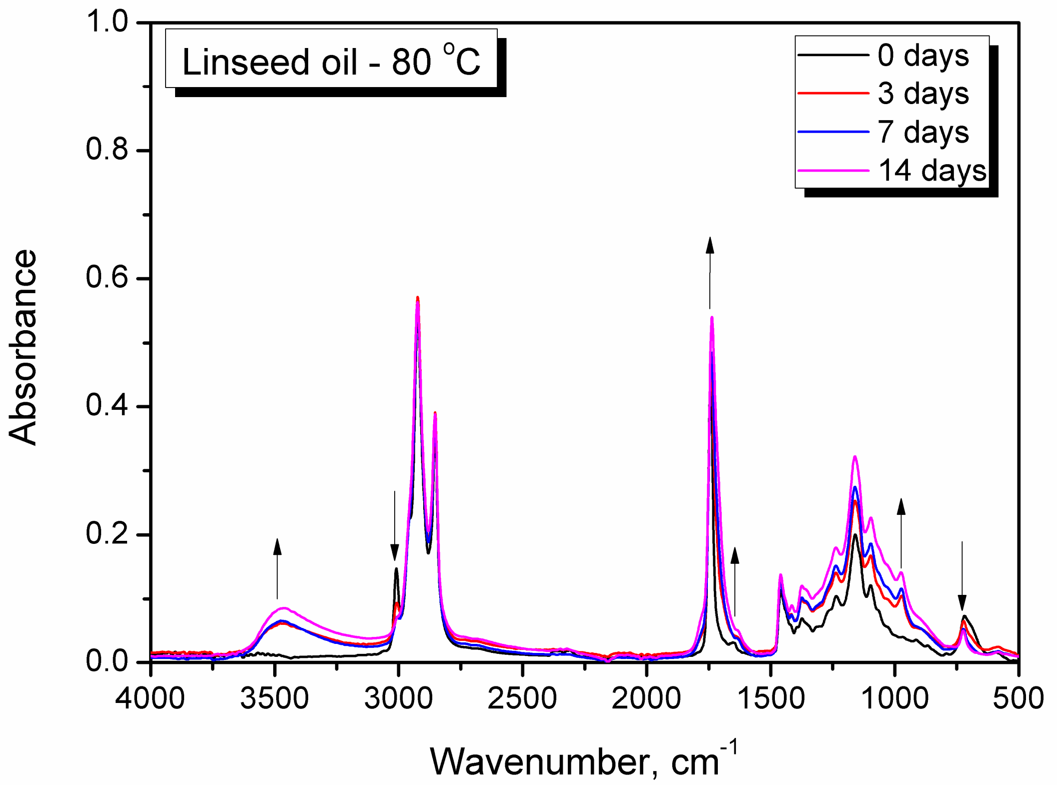

Figure S4. FTIR spectra of linseed oil subjected to thermooxidation at 80 °C.

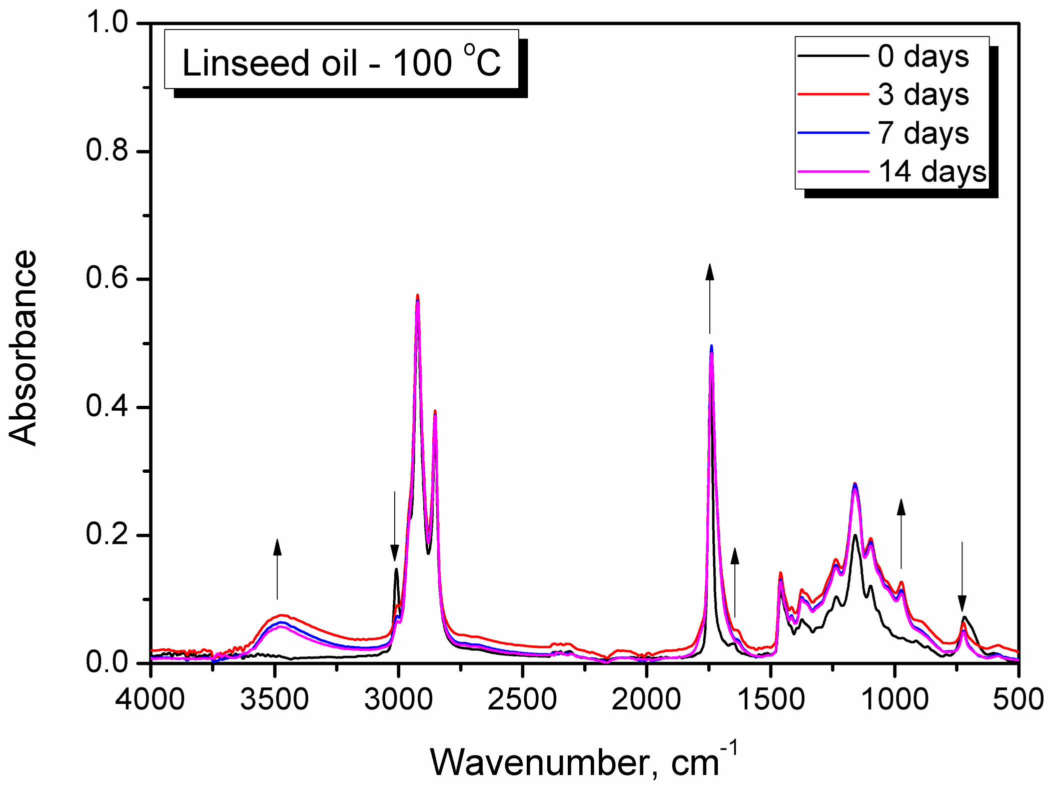

Figure S5. FTIR spectra of linseed oil subjected to thermooxidation at 100 °C.

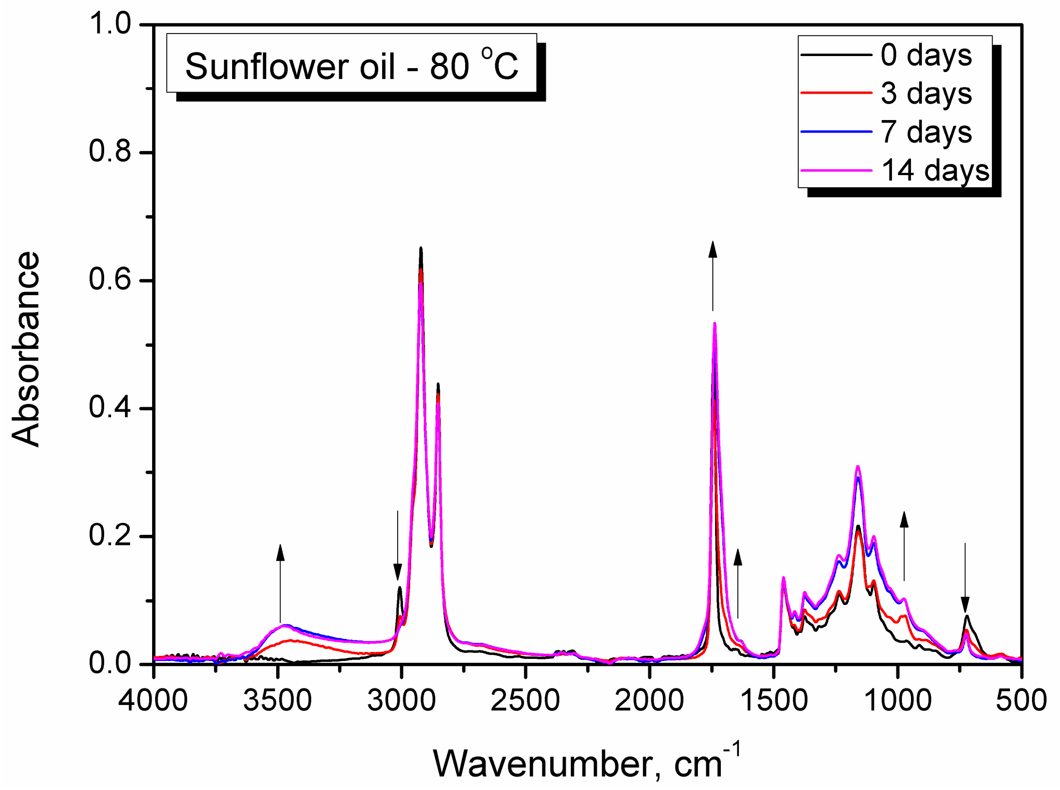

Figure S6. FTIR spectra of sunflower oil subjected to thermooxidation at 80 °C.

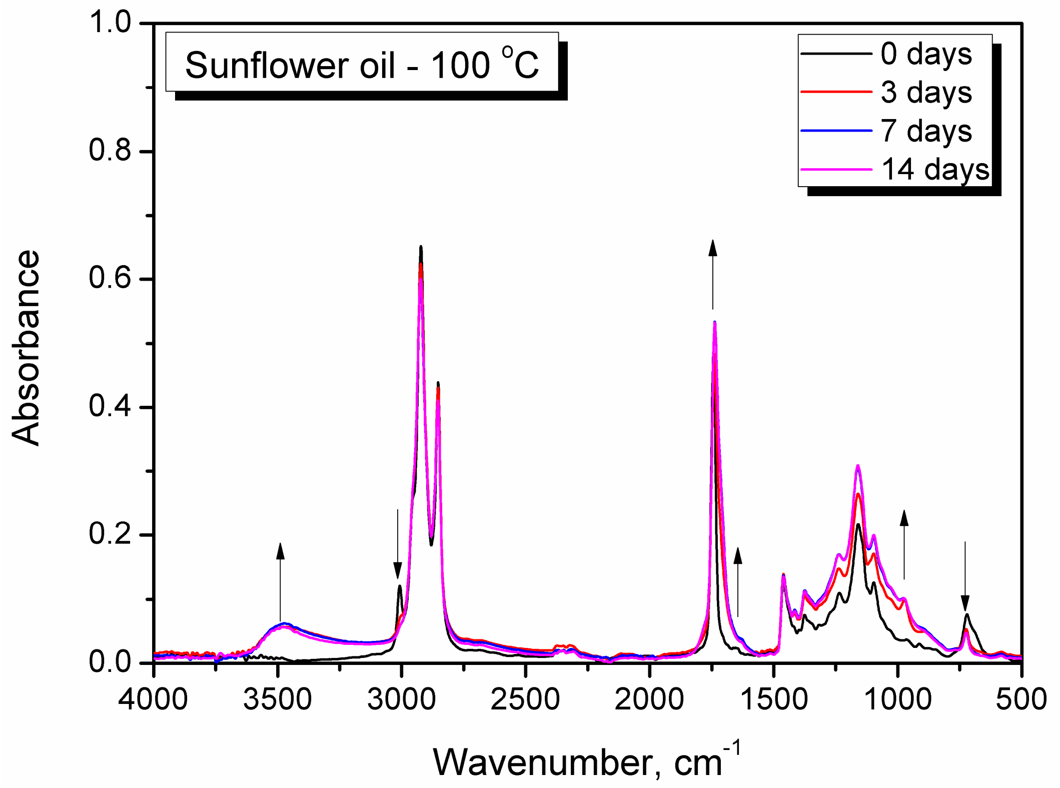

Figure S7. FTIR spectra of sunflower oil subjected to thermooxidation at 100 °C.

https://doi.org/10.3390/foods15050860

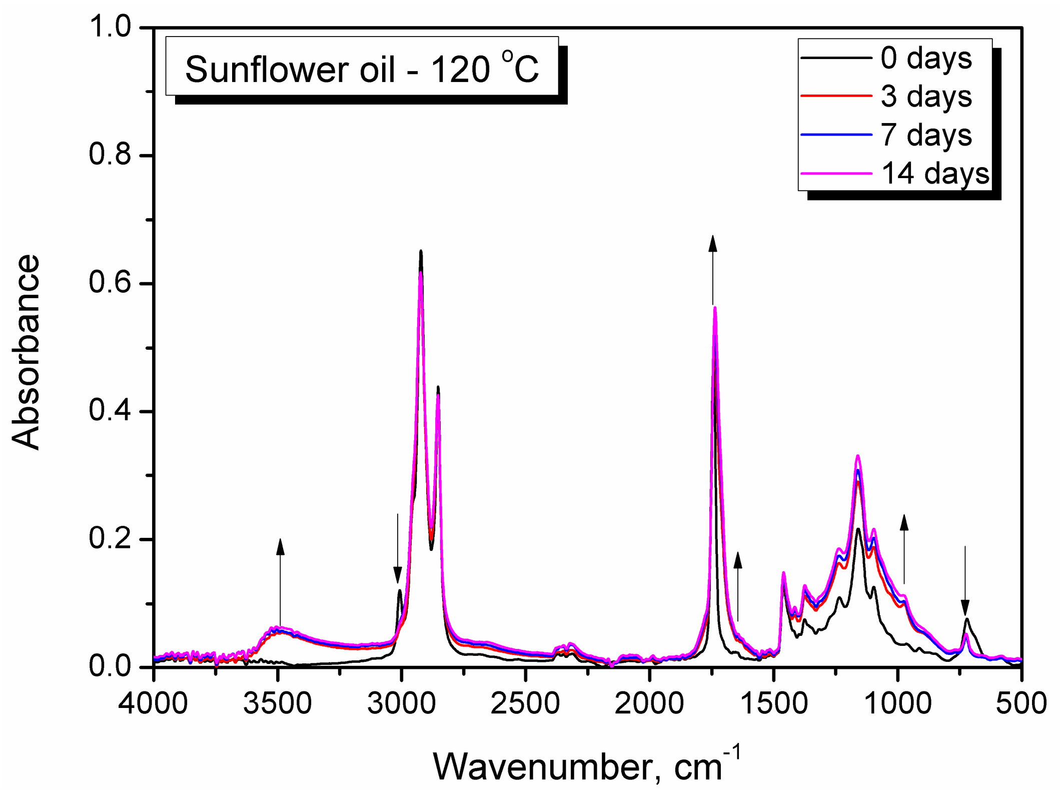

Figure S8. FTIR spectra of sunflower oil subjected to thermooxidation at 120 °C.

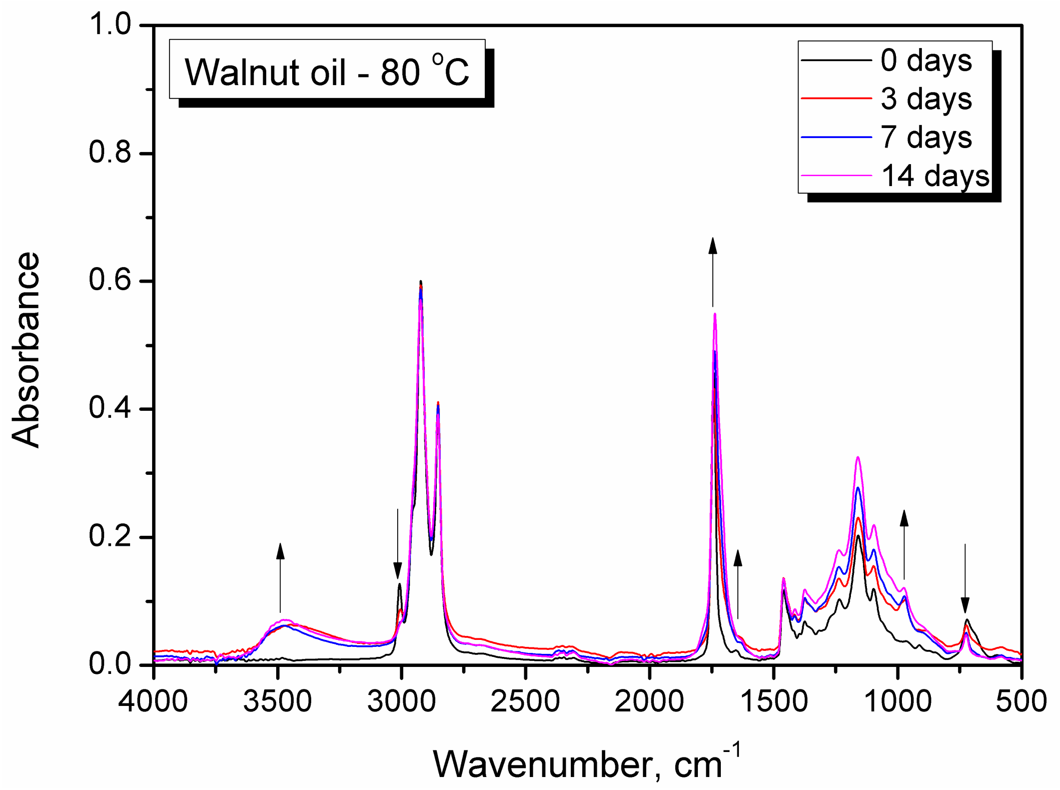

Figure S9. FTIR spectra of walnut oil subjected to thermooxidation at 80 °C.

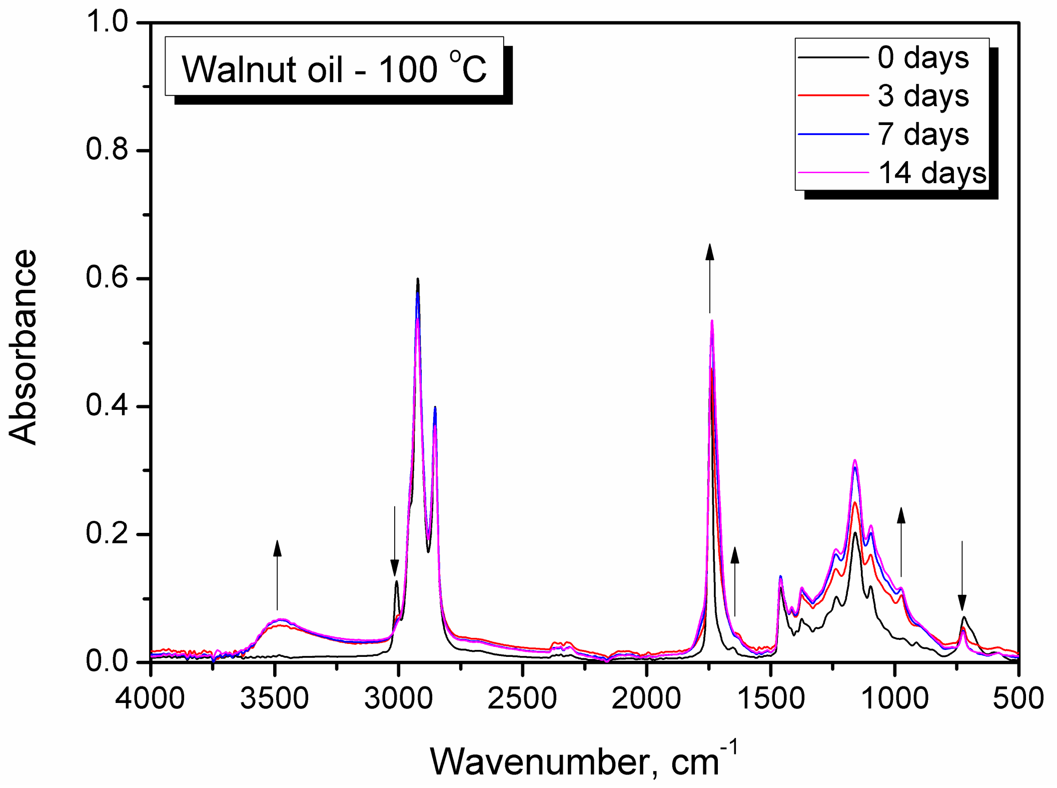

Figure S10. FTIR spectra of walnut oil subjected to thermooxidation at 100 °C.

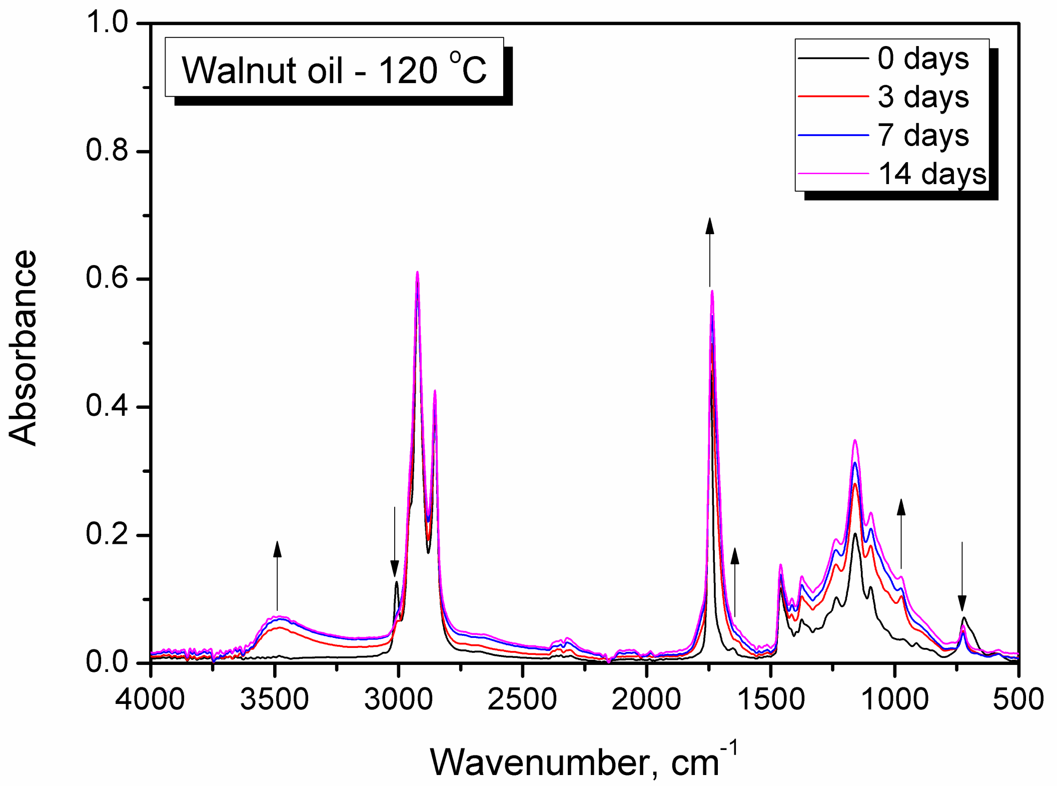

Figure S11. FTIR spectra of walnut oil subjected to thermooxidation at 120 °C.

https://doi.org/10.3390/foods15050860

S3. Additional results from differential scanning calorimetry

Table S3. Thermal parameters obtained from DSC for edible oils before and after 3, 7, and 14-day exposure to elevated temperature (80, 100, 120 °C) in oxidative atmosphere; T_M – melting temperature; T_C – crystallization temperature.

Oil type	Temperature	Aging time	T_{M1}	T_{M2}	T_{M3}	T_{C1}	T_{C2}	T_{C3}
	[°C]	[Days]	[°C]					
Hazelnut	-	0	-4.7	-	-	-49.0	-37.3	-20.8
	120	3	-10.8	5.1	-	-18.3	-42.0	-
		7	-34.6	-2.7	-	-25.9	-	-
		14	-34.5	-4.0	-	-28.8	-	-
Linseed	-	0	-37.6	-28.7	-12.2	-42.2	-19.2	-
	80	3	-21.8	8.1	-	-36.8	-21.6	-
		7	-21.4	-8.5	-	-38.2	-22.3	-
		14	-4.2	-	-	-39.8	-23.2	-
	100	3	-5.2	-	-	-22.8	-	-
		7	-8.3	-	-	-37.0	-24.2	-
		14	-4.7	-	-	-24.1	-0.5	-
	120	3	-6.8	-	-	-22.5	-	-
		7	-5.9	-	-	-22.5		
		14	-1.5	-	-	-	-	-
Peanut	-	0	-3.8	-	-	-48.3	-6.9	-
	120	3	-25.9	-0.2	-	-26.2	-5.3	-
		7	-39.5	-21.3	-4.0	-22.9	-4.1	-
		14	-32.4	-9.6	8.4	-22.8	-6.9	-
Sesame	-	0	-54.3	-31.6	-19.1	-38.8	-14.0	-
	120	3	-51.0	-30.5	-7.4	-34.0	-15.7	-
		7	-33.2	-0.3	-	-35.2	-17.9	-
		14	-34.9	-17.3	-1.9	-37.6	-19.9	-
Sunflower	-	0	-33.1	-24.0	-10.2	-43.3	-20.6	-
	80	3	-38.7	-14.3	-	-43.6	-27.4	-
		7	-37.3	-16.9	-8.2	-38.9	-25.9	-
		14	-33.4	-9.4	-	-26.9	-	-
	100	3	-45.5	-22.1	-8.4	-42.2	-24.3	-
		7	-32.7	-7.9	-	-28.6	-	-
		14	-24.6	-7.1	-	-29.9	-	-
	120	3	-36.9	-8.0	-	-39.7	-27.0	-
		7	-32.2	-7.8	-	-29.2	-	-
		14	-35.0	-8.5	-	-28.2	-	-
Walnut	-	0	-30.8	-	-	-46.5	-25.1	-
	80	3	-42.3	-19.3	-	-39.3	-36.1	-
		7	-36.7	-19.0	-	-16.2	-	-
		14	-36.0	-14.9	-	-39.6	-20.7	-
	100	3	-42.1	-18.6	-	-36.7	-	-
		7	-39.7	-16.5	-	-38.6	-	-
		14	-11.2	-	-	-19.6	-	-
	120	3	-42.9	-14.9	-	-37.4	-	-
		7	-40.1	-14.8	-	-36.2	-	-
		14	-14.5	-	-	-20.2	-	-

https://doi.org/10.3390/foods15050860

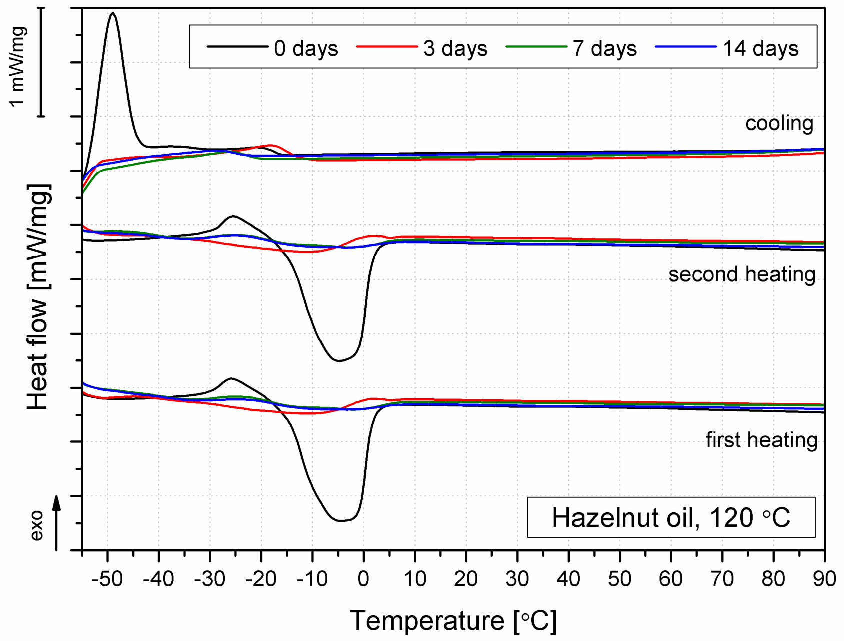

Figure S12. DSC heating and cooling curves of hazelnut oil subjected to 120 °C at 0, 3, 7, and 14 days.

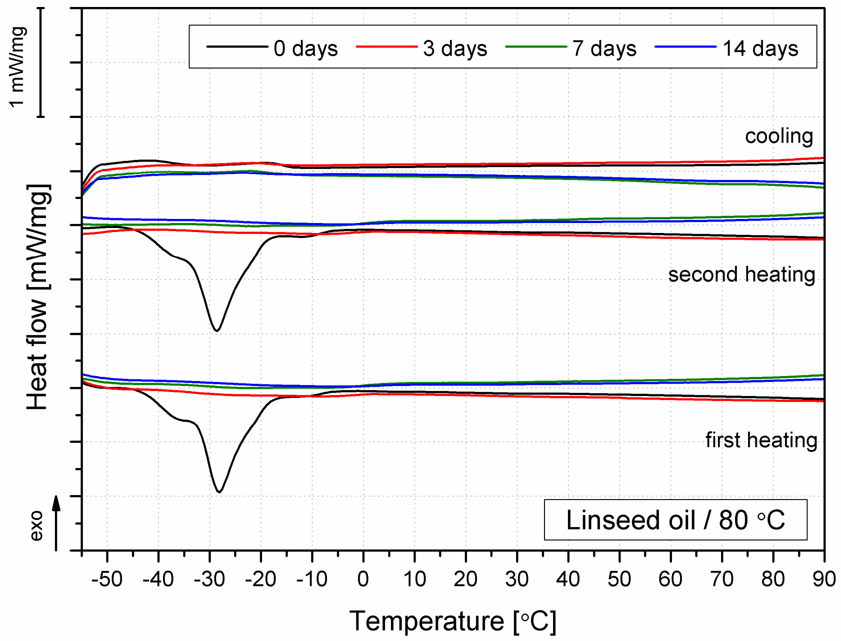

Figure S13. DSC heating and cooling curves of linseed oil subjected to 80 °C at 0, 3, 7, and 14 days.

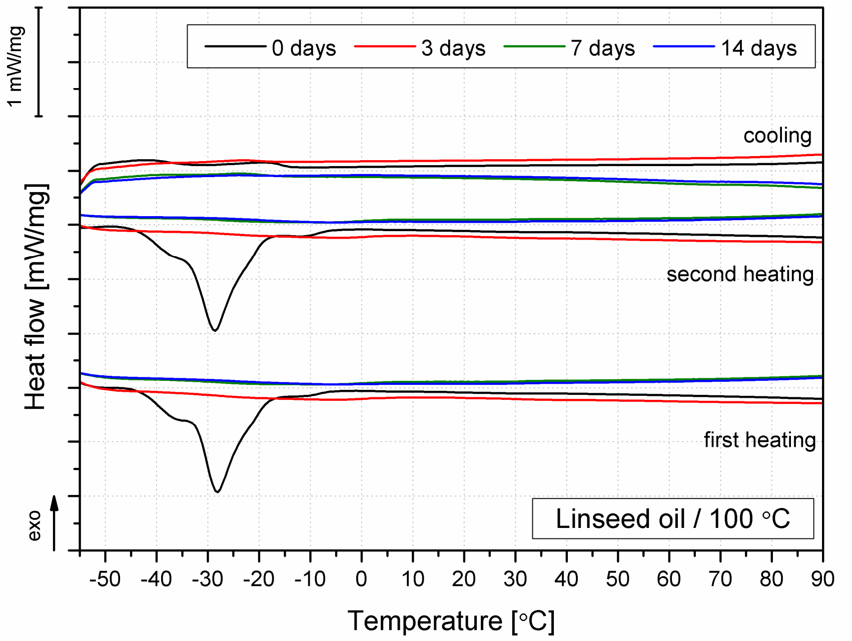

Figure S14. DSC heating and cooling curves of linseed oil subjected to 100 °C at 0, 3, 7, and 14 days.

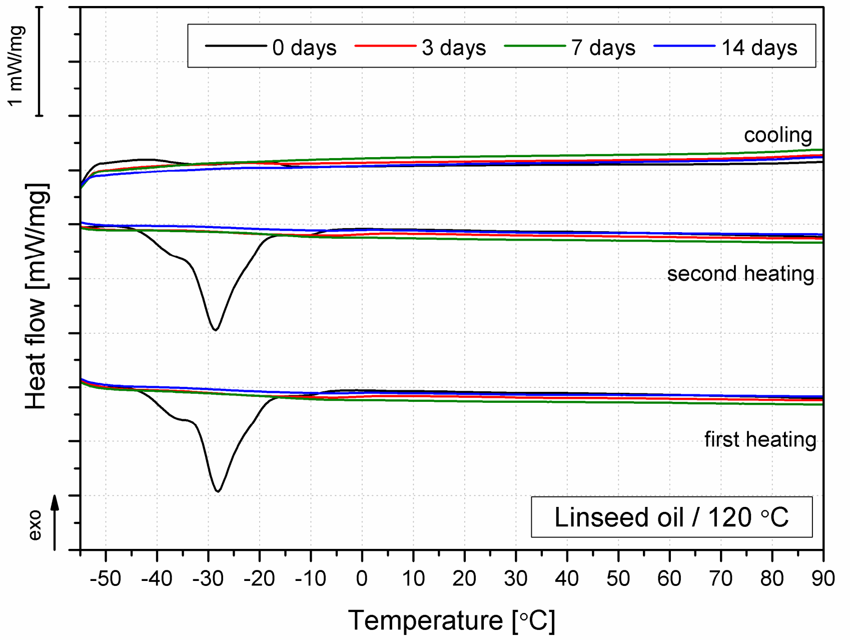

Figure S15. DSC heating and cooling curves of linseed oil subjected to 120 °C at 0, 3, 7, and 14 days.

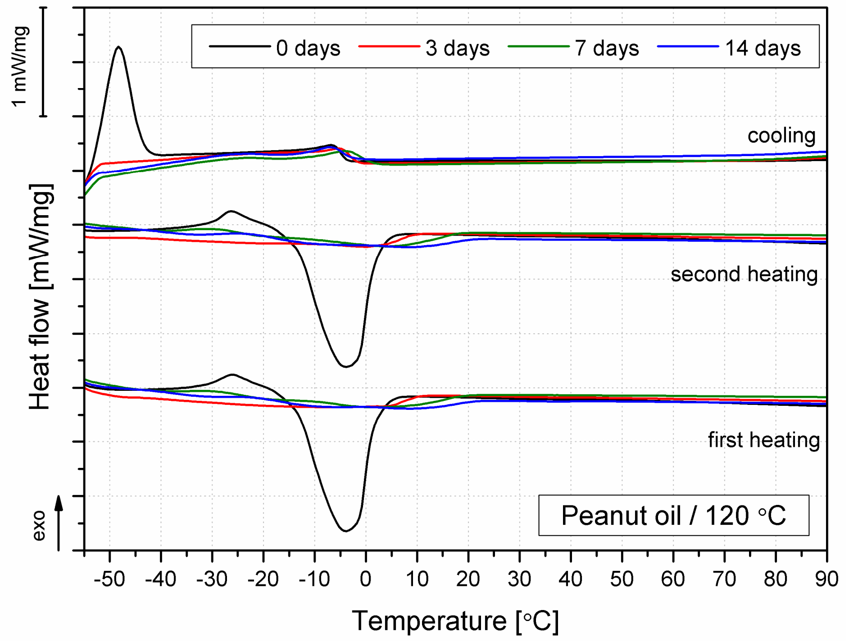

Figure S16. DSC heating and cooling curves of peanut oil subjected to 120 °C at 0, 3, 7, and 14 days.

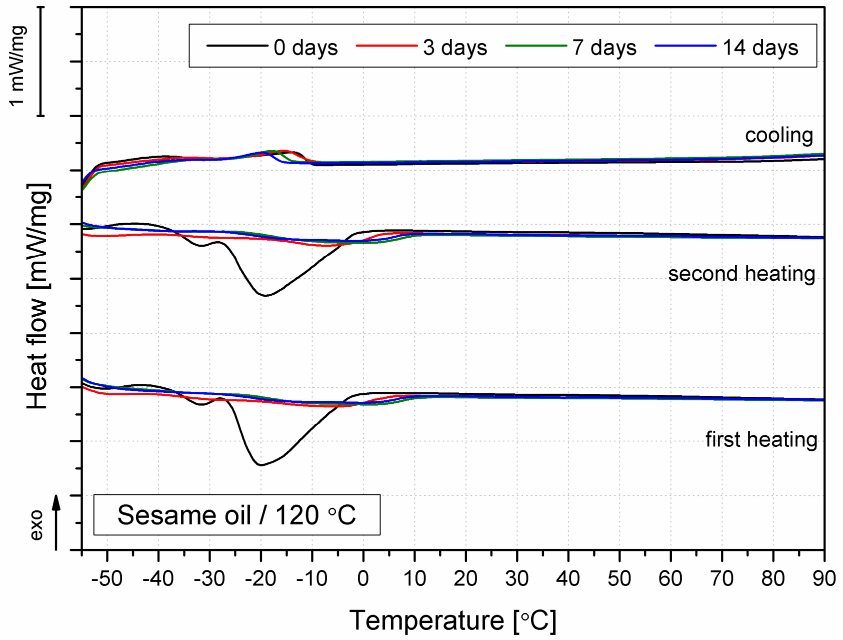

Figure S17. DSC heating and cooling curves of sesame oil subjected to 120 °C at 0, 3, 7, and 14 days.

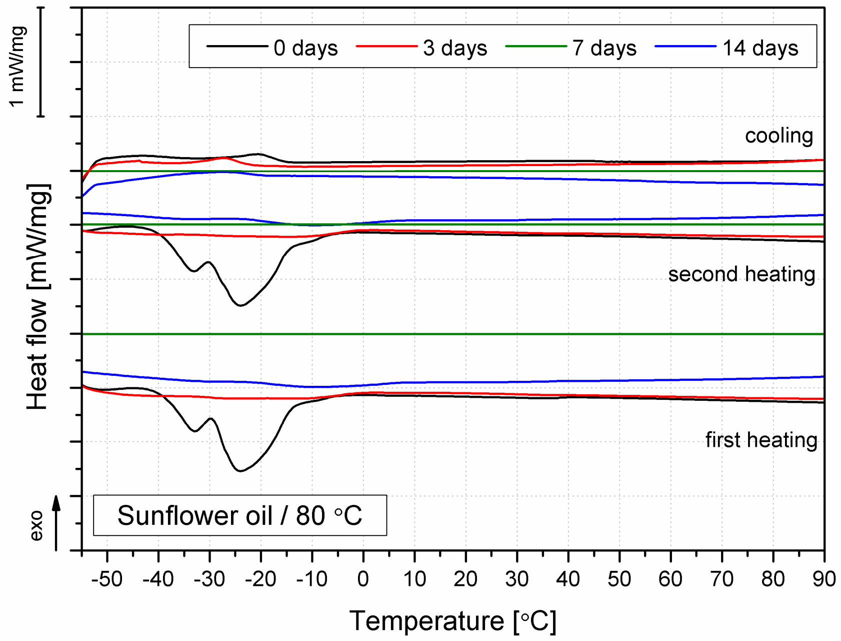

Figure S18. DSC heating and cooling curves of sunflower oil subjected to 80 °C at 0, 3, 7, and 14 days.

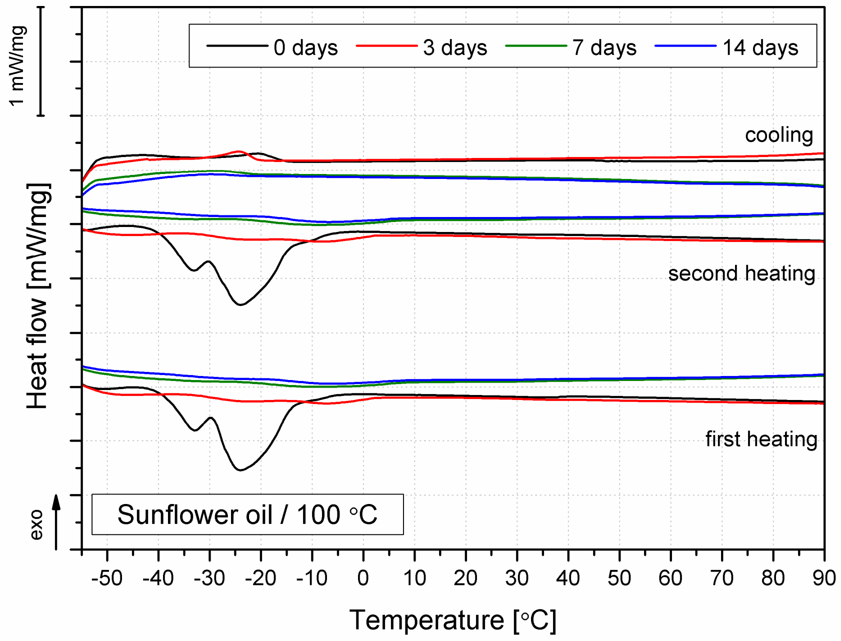

Figure S19. DSC heating and cooling curves of sesame oil subjected to 100 °C at 0, 3, 7, and 14 days.

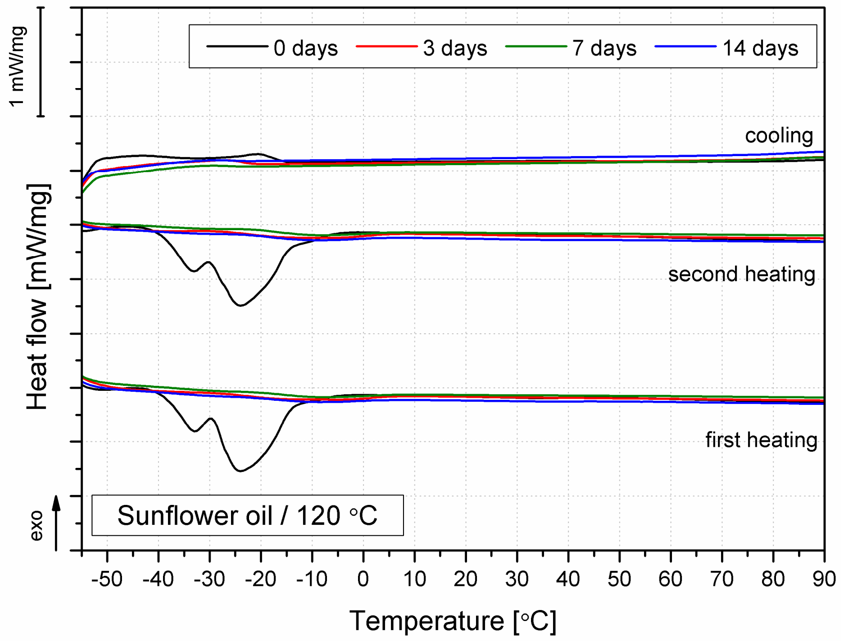

Figure S20. DSC heating and cooling curves of sunflower oil subjected to 120 °C at 0, 3, 7, and 14 days.

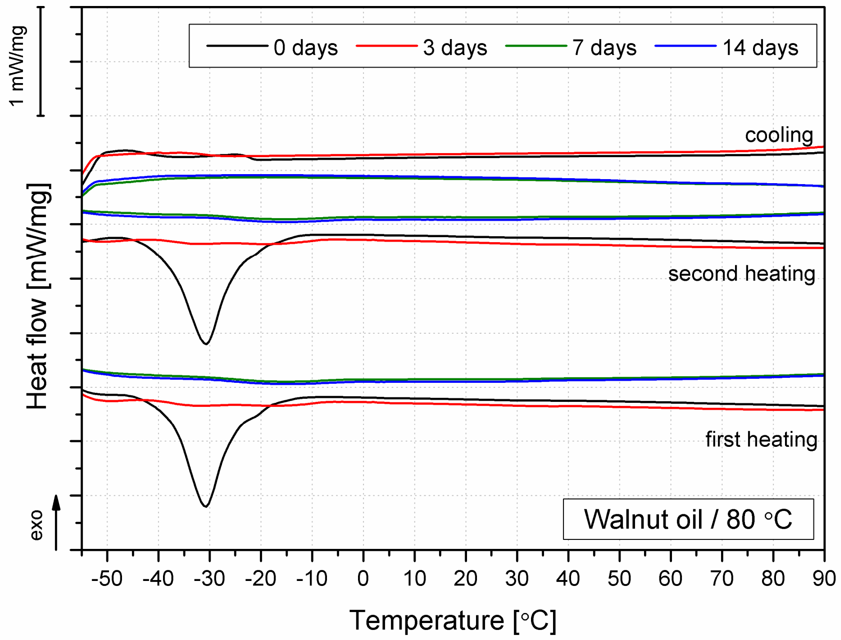

Figure S21. DSC heating and cooling curves of walnut oil subjected to 80 °C at 0, 3, 7, and 14 days.

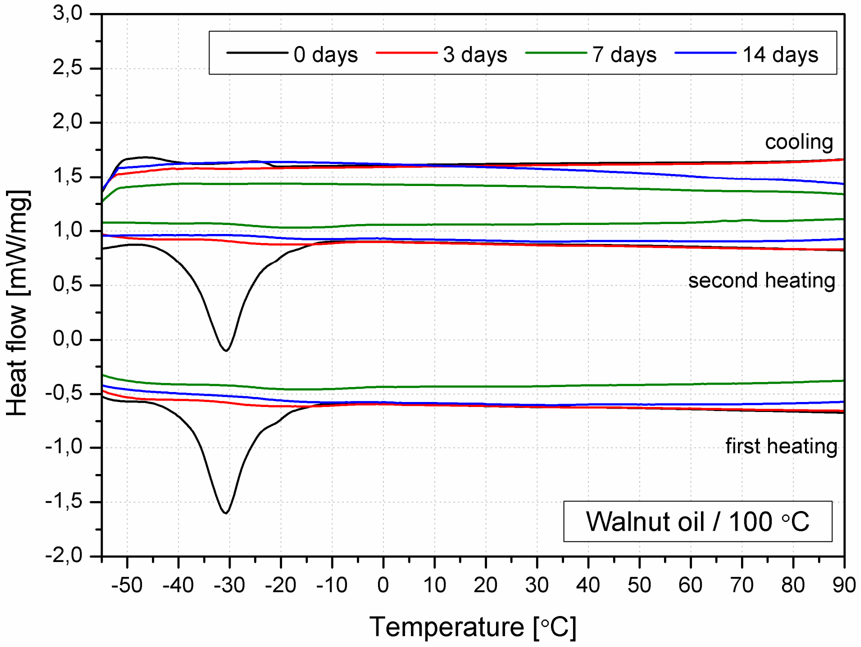

Figure S22. DSC heating and cooling curves of walnut oil subjected to 100 °C at 0, 3, 7, and 14 days.

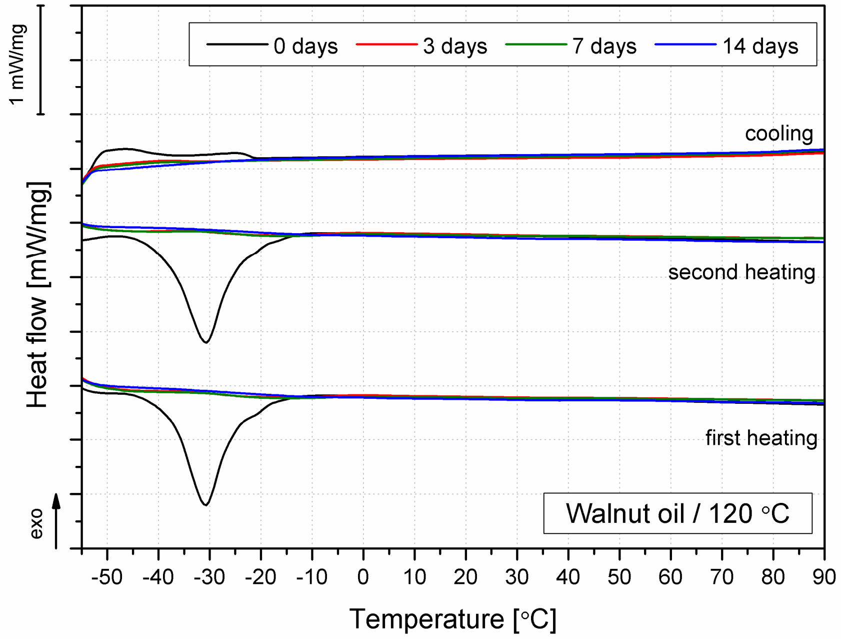

Figure S23. DSC heating and cooling curves of walnut oil subjected to 120 °C at 0, 3, 7, and 14 days.

https://doi.org/10.3390/foods15050860
